# Nanoparticle-Loaded Injectable Hydrogel Alleviates Titanium Particle-Induced Osteolysis by Disrupting GATA6/DDX3X-Mediated Macrophage Inflammation

**DOI:** 10.34133/bmr.0353

**Published:** 2026-04-16

**Authors:** Sipeng Lin, Taihe Liu, Qi Zhu, Zhuji Ouyang, Yifan Yu, Haopeng Sun, Changchuan Li, Shixun Li, Chenhao Pan, Wing Cheuk Ko, Haoxian Liu, Jin Liu, Shuangxing Li, Jinchang Chen, Shaojian Wu, Jichao Ye, Liangbin Gao, Yue Ding

**Affiliations:** ^1^ Department of Orthopedics, Shenshan Medical Center, Sun Yat-sen Memorial Hospital, Sun Yat-sen University, Shanwei, Guangdong 516621, P. R. China.; ^2^ Department of Orthopedic Surgery, Sun Yat-sen Memorial Hospital, Sun Yat-sen University, Guangzhou, Guangdong 510000, P. R. China.; ^3^ Guangdong Provincial Key Laboratory of Cancer Pathogenesis and Precision Diagnosis and Treatment, Shanwei, Guangdong 516621, P. R. China.

## Abstract

Aseptic loosening (AL) represents the primary cause of joint arthroplasty failure, which is predominantly triggered by chronic inflammatory reactions to prosthetic wear particles, with macrophages serving as the central effector cells. Accumulating evidence indicates that the crosstalk between endoplasmic reticulum stress and mitochondrial stress exacerbates macrophage-mediated inflammation; however, the core molecular regulators orchestrating this pathological cascade remain elusive. Herein, we investigated the functional role of the GATA6/DDX3X (DEAD-box helicase 3 X-linked) axis in titanium particle (TiP)-induced macrophage inflammatory responses and further explored the therapeutic potential of nanoparticle-loaded injectable hydrogels for AL. Specifically, a si-DDX3X-loaded nanoparticle hydrogel (si-DDX3X NPs@Hy) was fabricated and characterized, and its therapeutic efficacy was evaluated in vivo. Our results demonstrated that DDX3X expression was significantly up-regulated in both TiP-stimulated bone marrow-derived macrophages and periprosthetic tissues obtained from AL patients. Functional assays revealed that DDX3X promoted mitochondria–endoplasmic reticulum interplay, which in turn facilitated NLRP3 inflammasome assembly and subsequent interleukin-1β secretion. Mechanistically, GATA6 directly bound to the transcription start site of the DDX3X gene, thereby suppressing its transcriptional expression and abrogating DDX3X-mediated proinflammatory effects. The synthesized si-DDX3X NPs@Hy exhibited favorable physicochemical properties; local administration of this hydrogel markedly attenuated TiP-induced calvarial osteolysis in mice, accompanied by reduced osteoclastogenesis, proinflammatory cytokine production, and M1 macrophage polarization in the lesion microenvironment. Collectively, this study identifies the GATA6/DDX3X axis as a pivotal regulator of TiP-driven macrophage inflammation and validates the si-DDX3X NPs@Hy as a promising therapeutic strategy for targeting wear particle-induced inflammation, which holds great potential for improving the long-term prognosis of joint arthroplasty.

## Introduction

Arthroplasty stands as a primary surgical intervention for advanced joint disorders—most notably osteoarthritis and rheumatoid arthritis—delivering substantial therapeutic benefits by restoring joint function and alleviating pain [1]. However, with the rising incidence of arthroplasty procedures globally, associated complications have become increasingly prominent, among which aseptic loosening (AL) remains the leading cause of joint replacement failure [2]. This pathological process is primarily driven by chronic inflammatory responses triggered by long-term exposure to prosthetic wear particles (e.g., titanium debris) shed from the implant surface [3]. Over time, this persistent inflammation culminates in periprosthetic bone resorption and implant instability, often compelling revision surgery—an invasive procedure associated with high morbidity and healthcare costs [4]. Clinical data confirm that AL accounts for the majority of joint revision cases, with its pathogenesis involving intricate crosstalk between immune inflammation, dysregulated bone metabolism, and aberrantly activated signaling pathways [3,5]. Thus, elucidating the molecular mechanisms underlying AL and developing targeted therapeutic strategies are imperative to improving arthroplasty outcomes.

Macrophages occupy a central role in the pathophysiology of AL. Upon phagocytosis of wear particles, macrophages undergo activation and secrete copious amounts of proinflammatory cytokines, including tumor necrosis factor-α (TNF-α) and interleukin-1β (IL-1β) [6]. These cytokines accelerate bone resorption by promoting osteoclast differentiation and activation; concurrently, sustained activation of macrophage inflammatory signaling fosters the formation and maintenance of a proinflammatory microenvironment, which further amplifies AL progression [7]. Consequently, modulating macrophage-driven inflammation has emerged as a promising therapeutic avenue for mitigating AL.

Recent studies have highlighted that endoplasmic reticulum stress (ERS) and mitochondrial stress (MS) are critical drivers of macrophage inflammatory responses and M1 polarization (the proinflammatory macrophage subtype) [8,9]. In macrophages, ERS induced by stimuli such as lipopolysaccharide (LPS) regulates the expression of ERS sensor proteins—including inositol-requiring enzyme 1 (IRE1), protein kinase receptor-like ER kinase (PERK), and activating transcription factor 6 (ATF6)—thereby activating downstream inflammatory pathways and exacerbating macrophage-mediated inflammation [10]. Similarly, LPS-induced MS triggers the release of mitochondrial signaling molecules (e.g., reactive oxygen species [ROS] and mitochondrial DNA), which activate key inflammatory cascades (e.g., NLRP3 inflammasome, NF-κB, and mitogen-activated protein kinase [MAPK] pathways) and promote M1 macrophage polarization [11]. Notably, ERS and MS exhibit extensive crosstalk: ERS elevates mitochondrial intracellular calcium concentrations, disrupting mitochondrial homeostasis and inducing oxidative stress; conversely, MS reduces intracellular ATP levels and enhances ROS production, thereby triggering ERS [12,13]. This bidirectional interplay amplifies inflammatory responses in macrophages, further contributing to AL pathogenesis.

DDX3X (DEAD-box helicase 3 X-linked), a multifunctional RNA helicase, is involved in diverse RNA metabolic processes, including RNA splicing, translational regulation, and posttranscriptional modification [14]. Through high-throughput sequencing and bioinformatics analyses, we identified DDX3X as a key regulator of titanium particle (TiP)-induced macrophage inflammatory responses. Emerging evidence links DDX3X to multiple inflammatory signaling pathways: in macrophages, DDX3X modulates the production and secretion of proinflammatory cytokines by regulating NF-κB activation and NLRP3 inflammasome assembly [15,16]. Furthermore, recent studies suggest that DDX3X may influence the function of endoplasmic reticulum (ER)–mitochondria contact sites mitochondria-associated membranes (MAMs)—specialized subcellular structures critical for maintaining cellular homeostasis and orchestrating inflammatory stress responses [17,18]. These observations imply that DDX3X could play a pivotal role in TiP-induced macrophage inflammation by regulating ERS and mitochondrial function. However, the precise role of DDX3X in AL and its underlying molecular mechanisms remain incompletely understood.

GATA6, a highly conserved transcription factor of the GATA family, is broadly expressed in mammals and participates in a spectrum of cellular processes, including cell cycle regulation, proliferation, differentiation, and inflammation [19–21]. GATA6 is essential for the development and functional maintenance of multiple organs: for example, in intestinal epithelial cells, it modulates gut inflammation, while in the immune system, it regulates macrophage development and function [22,23]. Recent studies have demonstrated that under LPS stimulation, GATA6 mitigates inflammatory responses by inhibiting IL-1β and TNF-α production via the prostacyclin (PGI2)/IL-10 pathway [24]; additionally, in septic mouse models, GATA6 alleviates macrophage inflammation by suppressing ERS-mediated caspase-12 activation and NF-κB signaling [25]. These findings suggest that GATA6 may act as a negative regulator of macrophage inflammation, potentially interacting with DDX3X to modulate AL progression.

In recent years, orthopedics has witnessed rapid advancements in nanoparticle (NP) and injectable hydrogel technologies [26,27]. NPs, characterized by excellent biocompatibility and efficient drug delivery capacity, have been widely applied in targeted therapies and tissue engineering [28,29]. Injectable hydrogels, with their favorable biomechanical properties and localized drug release profiles, have emerged as promising tools for treating bone-related diseases [30,31]. By integrating the targeted drug delivery capabilities of NPs with the scaffold-like retention properties of hydrogels, NP-loaded injectable hydrogels enhance drug localization at the lesion site, prolong therapeutic efficacy, and minimize systemic side effects [32]. In orthopedic applications, these composite materials have shown potential for bone repair, cartilage regeneration, and the treatment of inflammatory bone disorders [33,34].

In summary, AL severely compromises the long-term success of arthroplasty, with macrophage-derived cytokines serving as central drivers of its pathogenesis. DDX3X and its upstream transcription factor GATA6—critical regulators of macrophage function—represent novel therapeutic targets for AL. Concurrently, NP-loaded injectable hydrogels, with their superior physicochemical properties and targeted drug delivery potential, offer a promising treatment modality. In this study, we developed a novel therapeutic strategy based on NP-loaded injectable hydrogels to specifically inhibit DDX3X-mediated macrophage inflammatory signaling, thereby reducing proinflammatory cytokine release and mitigating periprosthetic bone resorption. Our findings provide new mechanistic insights into the role of the GATA6/DDX3X axis in AL and offer theoretical and practical foundations for developing more effective therapeutic approaches to improve arthroplasty outcomes.

## Materials and Methods

### Clinical samples

The synovial membranes of the AL group were collected from 7 patients, including 4 males and 4 females with an average age of 65.4 ± 3.2 years, who accepted prosthesis revision surgery on account of AL with a mean duration of 15.25 ± 2.5 years. The synovial membranes of the control group were collected from 8 patients, including 4 males and 4 females with an average age of 66.8 ± 8.6 years, who accepted total hip arthroplasty on account of femoral head necrosis (FHN). The study was approved by the Ethics Committee at Shenshan Medical Center, SunYat-sen Memorial Hospital, Sun Yat-sen University ([2025-SSKY] Ethic Record No. 088).

### Immunohistochemistry assay and tissue immunofluorescence

Synovial membranes were put in 4% paraformaldehyde (PFA) for 12 h, then sequentially dehydrated in xylene and embedded in 54 °C paraffin for sectioning. The mice calvariae were employed in 4% PFA for fixation overnight and further decalcified by 10% EDTA (pH, 7.4) (E1171, Solarbio) for 21 d, then blocked with 54 °C paraffin and sectioned.

The tissues sections (3 μm) were heated at 60 °C for 2 h, then deparaffinized with xylene and rehydrated. Then, 0.1% Triton X-100 (9002-93-1, Sigma-Aldrich) was used for permeabilization and pepsin (ZLI-9013, ZSGB-BIO) was performed for antigen retrieval. Subsequently, the sections were blocked with 1% bovine serum albumin (BSA) for 1 h and then incubated with primary antibody for 2 h at 37 °C. Secondary antibodies conjugated with horseradish peroxidase (Goat anti Rabbit, ab6721, Abcam) were incubated at 37 °C for 30 min. After staining with DAB (P0203, Beyotime Biotechnology) and hematoxylin (Sigma-Aldrich) in sequence, the sections were observed under a Leica biomicroscope (DM2000, Leica). The intensity score (*I*_S_) was estimated by Bresalier’s evaluated system. The sections were estimated by 2 pathologists under blindness and 10 parts were randomly chosen to reduce the bias. Then the average *I*_S_ depended on the staining magnitude (0, stainless; 1, slight staining; 2, moderate staining; 3, strong staining) and the corresponding staining scale (F0 to F3). Eventually, the average *I*_S_ of each part was calculated with the following formula: ∑ (0 × F0 + 1 × F1 + 2 × F2 + 3 × F3).

The antibodies included IL-1β (Mouse, 3A6, Cell Signaling Technology), inducible nitric oxide synthase (iNOS; Rabbit, E1W4J, Cell Signaling Technology), DDX3X (Rabbit, ab271002, Abcam), and CD68 (Mouse, 66231-2-Ig, Proteintech).

### Titanium particles

TiPs (<20 μm, W08A030, Alfa Aesar) were diluted with sterilized water and then filtered by Isopore Membrane Filters (10/1.2/0.2 μm pore size, TCTP04700/RTTP04700/GTTP04700, Millipore) in filter holders. TiPs with an average diameter of 0.82 ± 0.12 μm were obtained and washed with 75% ethanol for 48 h. After the ethanol was thoroughly volatilized, the TiPs were weighed and further sterilized by ethylene oxide. TiPs were diluted into 9 × 10^−3^ g ml^−1^ with phosphate-buffered saline (PBS) for in vitro experiments. Then, the limulus amebocyte lysate assay (EC80545, Bioendo) was used to confirm that the TiPs were sterilized.

### Cell culture

The femurs and tibias from C57BL/6J male mice aged 8 weeks were isolated for bone marrow-derived macrophage (BMDM) extraction under sterilized conditions. The BMDMs were cultivated for 5 to 7 d with the stimulation of macrophage colony-stimulating factor (M-CSF) (51112-MNAH, Sino Biological Inc.) and were cultured at 37 °C and 5% CO_2_. Previous studies have confirmed that sterilized particles could induce a substantial IL-1β secretion in LPS-pretreated BMDMs while sole particles merely led to IL-1β secretion. Before the stimulation of TiPs, BMDMs normalized by cell counters (Countstar BioTech) were seeded 1 d in advance and 100 ng ml^−1^ LPS (Sigma-Aldrich) was applied for priming.

### Real-time PCR and chromatin immunoprecipitation-qPCR analysis

The RNAiso Plus kit (9109, TaKaRa Biotechnology) was performed to isolate the total RNA of macrophages. PrimeScript RT Master Mix (RR036D, TaKaRa Biotechnology) was used to convert RNA to cDNA. Then, quantitative real-time polymerase chain reaction (qRT-PCR) was conducted in a LightCycler 96 Real-Time PCR System (Roche Molecular Systems, Inc.) using SYBR Green Mix (Yeasen Biotech Co., Ltd.). The 2^−ΔΔCt^ formula was performed to quantify the gene expression. The primers are shown in Table S1.

Chromatin immunoprecipitation (ChIP) was conducted with the ChIP Assay Kit (P2078, Beyotime) and subsequent qPCR details were listed above.

### ELISA analysis

Cell culture supernatants by TiP-induced BMDMs were collected. The inflammatory cytokine levels of IL-1β and interferon-β (IFN-β) were gauged with the instant enzyme-linked immunosorbent assay (ELISA) kit (EMC001b.96, EMC016.96, Neobioscience Technology Co., Ltd).

### Immunofluorescence staining

Following 15 min of fixation in 4% PFA, the cells were incubated for 15 min with 0.1% Triton X-100 at 20 °C and then gently shaken. Then, the cells were blocked with 1% BSA and incubated with IL-1β (Rabbit, A1112, Abclonal), p65 (Rabbit, 8242, Cell Signaling Technology), ATF6 (Rabbit, 24169-1-AP, Proteintech), NRF2 (Rabbit, 12721, Cell Signaling Technology), and IRF3 antibodies (Rabbit, ab68481, Abcam) overnight at 4 °C. The cells were disposed with secondary antibodies (Goat anti Rabbit, A32740; Goat anti Mouse, A32723, Thermo Fisher Scientific) at 20 °C for 1 h, then disposed with DAPI (HNFD-02, HelixGen Co, Ltd.) for nuclear counterstaining and observed with a Carl Zeiss confocal microscope.

### MitoTracker and ER tracker staining

The BMDMs stimulated by TiP were stained by MitoTracker Red and ER Tracker Green to analyze the mitochondrial injury and ER response. The BMDMs were incubated with the MitoTracker Red CMXRos (C1035, Beyotime) reagent or ER Tracker Green (C1042S, Beyotime) for 30 min after TiP stimulation. Next, the cells were counterstained with Hoechst 33342 (P0133, Beyotime) and then observed by fluorescence microscopy (LSM 710, Carl Zeiss).

### Western blot and coimmunoprecipitation

BMDMs were seeded in 6-well plates at a density of 2 × 10^5^ cells per well. After the primary stimulation of TiPs, BMDMs were lysed with the radio-immunoprecipitation assay (RIPA) buffer (9806S, Cell Signaling Technology) together with phenylmethylsulfonyl fluoride (PMSF) (P0100-1, Solarbio) and phosphatase inhibitor cocktails (CW2383, CWbiotech) to gain total protein. Nuclear and cytoplasmic lysates were obtained using the nuclear and cytoplasmic extraction kit (CWbiotech). Next, the concentration of protein was normalized by bicinchoninic acid protein assay (23225, Thermo Fisher Scientific), after which samples were separated and transferred. After that, the membrane containing protein was incubated in turn with TBS-Tween (CW0043S, CWbiotech) supplemented 5% BSA (room temperature, 1 h), primary antibody (4 °C, overnight), and secondary antibody (room temperature, 1.5 h). After primary and secondary antibody incubation, wash PVDF membrane with TBST (T1081, Solarbio) for 3 times, 5 min each. Then, the PVDF membrane was soaked into ECL substrate (WBULP-100ML, Millipore) and exposed in a digital image system (Gel Logic 2200Pro, Kodak).

A previous study had clearly illustrated the method of coimmunoprecipitation [35]. The experiment was conducted via the Pierce Classic Magnetic co-IP kit (88804, Thermo Fisher Scientific). Shortly, the cell lysis was incubated with anti-NLRP3 IP-class antibodies overnight in a 4 °C refrigerator. The antibody–antigen complex was mixed with Protein A/G magnetic beads for 1 h at room temperature. Wash beads with IP lysis buffer twice and ultrapure water once and mix beads with elution buffer for collecting supernatant. Next, the collected antigen–antibody complex was assessed with sodium dodecyl sulfate–polyacrylamide gel electrophoresis (SDS-PAGE) process.

The antibodies included p-PERK (Rabbit, 3179, Cell Signaling Technology), p-IRE1 (Rabbit, 3294, Cell Signaling Technology), β-Actin (Rabbit, 4970, Cell Signaling Technology), DDX3X (Rabbit, 8192, Cell Signaling Technology), IL-1β (Rabbit, 12242, Cell Signaling Technology), Caspase1 (Rabbit, 24232, Cell Signaling Technology), NLRP3 (Rabbit, 15101, Cell Signaling Technology), Cleaved-Caspase1 (Rabbit, 89322, Cell Signaling Technology), Apoptosis-associated Speck-like protein containing a CARD (ASC) (Rabbit, 67824, Cell Signaling Technology), p-IRF3 (Rabbit, 29047, Cell Signaling Technology), DRP1 (Rabbit, 8570, Cell Signaling Technology), FIS1 (Rabbit, 32525, Cell Signaling Technology), and GATA6 (Rabbit, 5851, Cell Signaling Technology).

### RNA-seq

Total RNA was extracted from the Trizol reagent kit (Invitrogen, Carlsbad, CA, USA)-lysed BMDMs stimulated with TiP for 4 h. RNA sample integrity was assessed using an Agilent 2100 Bioanalyzer (Agilent Technologies, Palo Alto, CA, USA), followed by validation via RNase-free agarose gel electrophoresis. Following ribosomal RNA depletion, remaining messenger RNAs and noncoding RNAs were enriched, fragmented by fragmentation buffer, and reverse-transcribed into cDNAs with random primers. cDNA modification involved end-repair, poly(A) tail addition, and ligation of Illumina sequencing adapters. Post-UNG (uracil-N-glycosylase) digestion, agarose gel electrophoresis was performed for size selection, followed by PCR amplification. Sequencing was conducted using Illumina HiSeq 4000, with adapter-containing reads and low-quality sequences (*Q* value ≤ 20) excluded. Clean reads were aligned to the mouse reference genome (GRCm38) using MapSplice and HTSeq for gene-level read count estimation. Differentially expressed genes (DEGs) were identified via the R package edgeR with significance criteria of false discovery rate (FDR) < 0.05 and log2(fold change) > 1. Gene Set Enrichment Analysis was performed using the online platform (http://software.broadinstitute.org/gsea), while Gene Ontology and Kyoto Encyclopedia of Genes and Genomes pathway analyses were conducted using the R package clusterProfiler.

### Flow cytometry

The BMDMs were trypsinized and collected by centrifugation at 1,000 rpm for 5 min. Then, the cells were fixed with the fixation buffer (00-8222-49, Thermo Fisher Scientific) and washed with Perm/Wash Buffer (554723, BD Biosciences). The antibody of macrophage polarization biomarkers iNOS or CD206 was added for 20 min of incubation at 20 °C avoiding illumination. Isotype controls were used as negative controls. Next, the cells were resuspended in 200 μl of PBS for each sample after washing 3 times, and then analyzed by flow cytometry (BD FACSVerse, BD Biosciences).

Data acquisition was performed using a flow cytometer, and all data were analyzed using FlowJo software. During data analysis, cell debris was first excluded based on forward scatter (FSC) and side scatter (SSC) properties. Doublets were subsequently removed using FSC-A versus FSC-H gating to obtain viable single-cell populations. Polarization subsets were then quantified based on iNOS^+^ (M1) and CD206^+^ (M2) expression within the gated BMDM population. The percentage of positive cells was calculated from independent biological replicates and expressed as mean ± SD.

### Micro-CT

In prior research, the micro-CT scanning and 3-dimensional (3D) reconstruction processes of calvaria were thoroughly described [35]. All calvarial samples were preserved using 4% PFA and scanned via a micro-CT imaging system (ZKKS-MCT-Sharp, Zhongke Kaisheng Medical Technology). The acquired data underwent subsequent quantitative analysis under conditions of 60 kV voltage and 667 μA current, with 3D model reconstruction performed using the supporting software (ZZKS-Micro-CT4.1). A 1 × 3 × 3 mm^3^ region of interest was then defined to measure parameters including bone mineral density (BMD), bone volume (BV)/total volume (TV) ratio, and the area of osteolysis.

### Animal surgery

For the construction of cranial osteolysis models and in vivo experiments, 10-week-old male C57B6/J mice were sourced from the Animal Laboratory of Sun Yat-sen University. Anesthesia and surgical procedures were in accordance with the guidelines of the Animal Ethical and Welfare Committee of Sun Yat-sen University.

Under anesthesia, a 10-mm midline sagittal incision was made on the mice's heads to expose the calvaria. A 0.5 × 0.5 × 0.3 cm^3^ gelatin sponge was positioned over Bregma for subsequent injection. The mice were randomized into 6 groups, each comprising 6 animals: (a) sham-operation group (Control); (b) TiP-treated control group (Ti); (c) NC + SN-NPs@Hy group: negative control (NC) lentivirus and si-NC NP-loaded injectable hydrogel cotreated group; (d) NC + SD-NPs@Hy group: NC lentivirus and si-DDX3X NP-loaded injectable hydrogel cotreated group; (e) SG+SN-NPs@Hy group: GATA6 knockdown (si-GATA6) lentivirus and si-NC NP-loaded injectable hydrogel cotreated group; and (f) SG+SD-NPs@Hy group: si-GATA6 lentivirus and si-DDX3X NP-loaded injectable hydrogel cotreated group.

All mice were euthanized on day 14 postsurgery, and their calvariae were harvested. The entire in vivo experiment was approved and executed following the institutional guidelines for laboratory animal care at Sun Yat-sen University.

### Lentivirus and siRNA transfection

Cyagen was responsible for constructing lentiviruses designed to overexpressing Gata6 (NCBI GeneID: 14465), with NC lentiviruses serving as control vectors. For stable transfection, lentiviruses (1×10^7^ TU/ml) were incorporated into complete culture medium for a 48-h transfection period, followed by puromycin selection to establish polyclonal stable-transfected cell lines.

GenePharma constructed the siRNA targeting DDX3X (NCBI GeneID: 13205), using NC siRNA as controls. After macrophages were plated and reached 60% confluence, siRNA complexes were formed with Lipofectamine LTX (15338100, Thermo Fisher Scientific) in OptiMEM (31985070, Thermo Fisher Scientific) and added to the cells for 24-h incubation. The transfection medium was then replaced with complete medium to proceed with subsequent experiments. The siRNA sequences are shown in Table S2.

### Dual luciferase reporter assay

To validate the regulatory effect of GATA6 on the DDX3X promoter, the pGL3-Basic plasmid harboring the DDX3X promoter was constructed by IGEBio. HEK293T cells were seeded in 12-well plates and cultured until reaching 80% confluence. Following transfection with the DDX3X promoter-containing plasmid, empty vectors and GATA6 overexpression plasmids were cotransfected at varying ratios. Luciferase activity was detected using the Firefly/Renilla Luciferase Assay Reagent with SYNERGY H1 (Bio-Tek), normalized by Renilla luciferase expression via the pRL-TK plasmid.

### Preparation of thermosensitive hydrogel carrying NPs

siRNA-loaded NPs were prepared using a classical double emulsion–solvent evaporation method, as previously described in our published work [36]. Briefly, si-DDX3X or negative control siRNA (si-NC) was reconstituted in diethyl pyrocarbonate (DEPC) water and complexed with polyamidoamine (PAMAM) at an N/P ratio of 15:1 to form siRNA/PAMAM complexes. Subsequently, 10 mg of carboxyl-terminated poly(lactic-co-glycolic acid) (PLGA-COOH) was dissolved in 500 μl of dichloromethane to obtain a homogeneous organic phase. Under ice-bath conditions, the siRNA/PAMAM complex solution was added dropwise to the PLGA solution under continuous stirring at 1,000 rpm to form the primary emulsion. This emulsion was then added dropwise into 4 ml of an aqueous phase containing 5% (w/v) polyvinyl alcohol under gentle stirring at room temperature for 4 h to allow solvent evaporation. Finally, NPs were collected by high-speed centrifugation (5 min) and washed twice with DEPC water to yield purified siRNA-NPs.

The thermosensitive hydrogel was prepared according to previously reported methods with minor modifications [37]. Briefly, hyaluronic acid (HA) was dissolved in DEPC water, and methacrylic anhydride (MA) was added dropwise under continuous stirring. The mixture was vortexed thoroughly and allowed to react overnight at 4 °C. The reaction product was purified using a 10,000 molecular weight cutoff dialysis membrane against 10 mmol/l NaCl buffer for 2 d to remove unreacted reagents, followed by sterile filtration and lyophilization to obtain methacrylated hyaluronic acid (HA-MA) powder.

The thermosensitive hydrogel was synthesized by polymerizing HA-MA in the presence of *N*,*N*,*N*′,*N*′-tetramethylethylenediamine, methyl methacrylate, and potassium persulfate at 20 °C under nitrogen for 24 h, followed by freeze-drying. For application, siRNA-NPs (10 mg/ml) were dissolved in double-distilled water to prepare a final 5% (w/v) thermosensitive hydrogel system, which was then thoroughly mixed to obtain the siRNA-NPs@hydrogel composite.

### Characterizations of NPs

The particle size distribution and zeta potential of the NPs were determined by dynamic light scattering (Brookhaven Instruments, USA). For transmission electron microscopy imaging, a droplet of NP suspension was placed onto a carbon-coated copper grid (300 mesh), air-dried at room temperature, and examined for morphological characterization. To quantify the encapsulation efficiency (EE%) and loading content (LC) of NPs, Cy5-labeled siRNA was incorporated into the NPs. The NP suspension was centrifuged at high speed to pellet the NPs, and the concentration of unencapsulated (free) siRNA in the supernatant was measured using a fluorescence spectrophotometer. The amount of encapsulated siRNA was determined by subtracting the mass of free siRNA in the supernatant from the total initial mass of siRNA added. For in vitro siRNA release kinetics, Cy5-siRNA-loaded NPs were suspended in PBS and gently stirred at constant temperature. At predetermined time intervals, aliquots were collected, and the NPs were pelleted by centrifugation and dissolved in dimethyl sulfoxide. The fluorescence intensity of Cy5-siRNA was then measured to evaluate the amount of siRNA released.

The siDDX3X loading content in different NP formulations (PLGA-siDDX3X, PAMAM-siDDX3X, and SD-NPs) was calculated using the aforementioned indirect method and expressed as micrograms of siRNA per milligram of NPs (μg siRNA/mg). This quantitative analysis was used to evaluate the siRNA loading capacity of different carrier systems. For hydrogel formulations used in the in vivo experiments, SD-NPs were uniformly dispersed in the hydrogel precursor solution prior to gelation. SD-NPs (0.8 mg) were incorporated into each hydrogel injection based on the measured loading content.

### Statistics

All experiments were replicated a minimum of 3 times to reduce potential bias. Data analysis was conducted using GraphPad Prism (version 9.3.0). The Kolmogorov–Smirnov test was employed to evaluate data normality. For pairwise comparisons between 2 groups, Student *t* test was used to determine statistical significance. In cases of multigroup comparisons with single or dual variables, 1-way analysis of variance (ANOVA) or 2-way ANOVA, respectively, were applied, followed by Bonferroni’s correction. A *P* value less than 0.05 was deemed statistically significant when contrasting intervention groups with control groups, indicated by *P < 0.05. All data are presented as the mean ± standard deviation (SD).

## Results

### Macrophage inflammatory responses, mitochondrial injury, and ERS were detected in synovial membranes and BMDMs

**Fig. 1.**
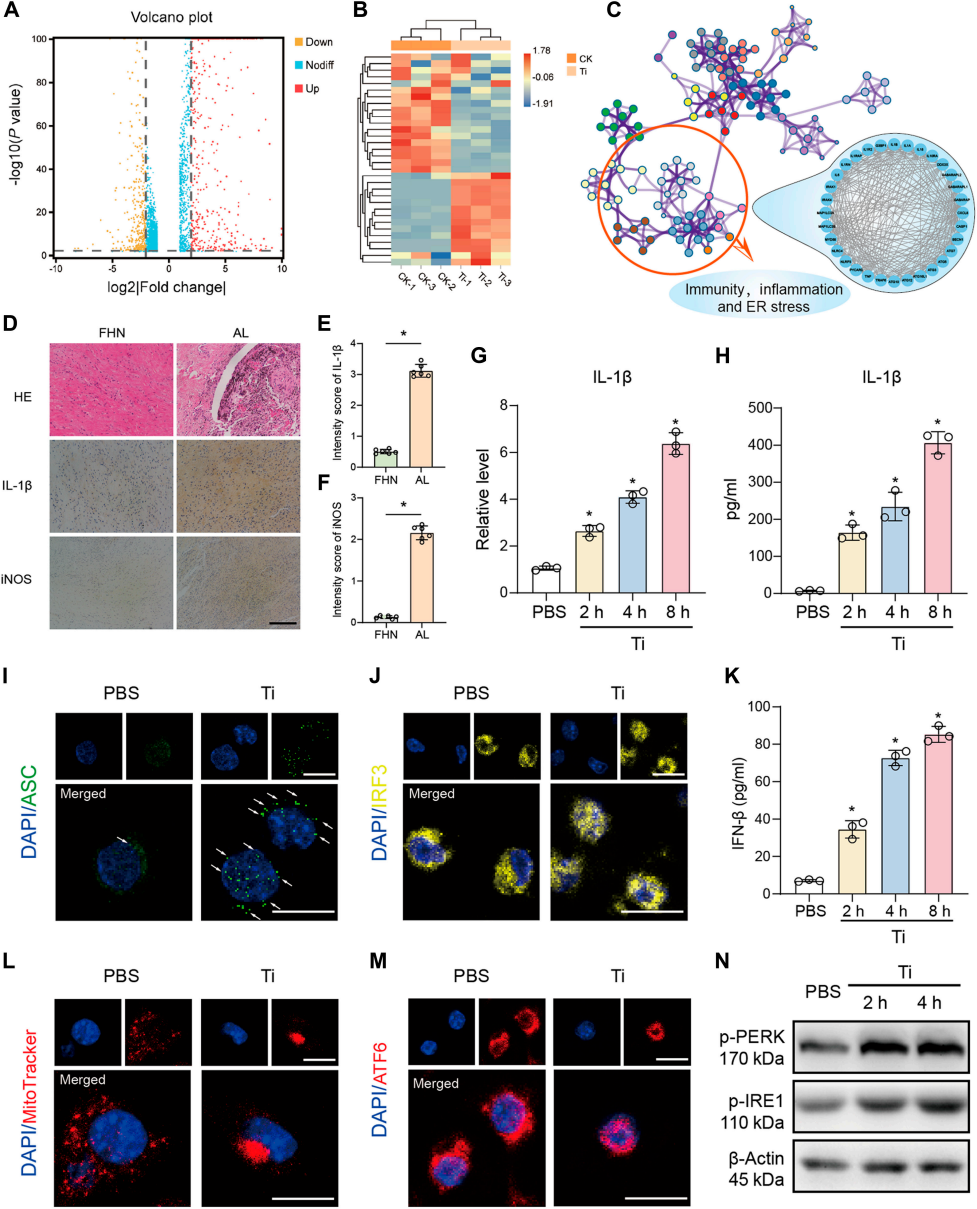
Titanium particles (TiPs) induced inflammatory cytokine production and cellular organelle imbalance. (A) Volcano plot of RNA-seq (|log_2_FC| < 1, *P* < 0.05). (B) Heatmap of differentially expressed genes (DEGs). CK stands for phosphate-buffered saline (PBS)-treated control group, and Ti means TiPs-stimulated group. (C) Enriched pathway network was generated by Metascape. Immunity, inflammation, and endoplasmic reticulum (ER) stress was annotated. (D) Histology staining of FHN (femoral head necrosis) and AL (aseptic loosening) synovium. HE (hematoxylin–eosin), tumor necrosis factor-α (TNF-α), and iNOS antibody were utilized. Scale bar, 500 μm. (E and F) Intensity score of TNF-α (E) and iNOS (F) immunohistochemical (IHC) staining (*n* = 6) were both higher in the AL group. (G and H) IL-1β mRNA (G) and secretion (H) level of macrophages stimulated by TiPs for 2, 4, and 8 h. (I and J) Cellular staining of ASC (I) and IRF3 (J) in PBS or TiP-challenged macrophages. Scale bar, 25 μm. (K) IFN-β secretion level of macrophages stimulated by TiPs for 2, 4, and 8 h. (L and M) Mitotracker (L) and ERS marker ATF6 (M) staining of TiP-induced macrophage and control group. Scale bar, 25 μm. (N) Immunoblots of p-PERK and p-IRE1 for PBS or TiP-challenged macrophages (2 and 4 h). β-Actin was used for loading control. Results displayed in the form of mean ± SD utilized two-tailed Student *t* test and 1-way analysis of variance (ANOVA) for calculating statistical significance. All experiments were triplicated at least. *P < 0.05.

To investigate global transcriptomic changes in macrophages upon TiP exposure, we performed RNA sequencing (RNA-seq) on TiP-challenged macrophages compared to PBS-treated controls. DEGs were visualized using a volcano plot (Fig. 1A) and heatmap (Fig. 1B). Functional enrichment analysis of DEGs via Metascape revealed a network of enriched pathways (Fig. 1C), with immune response, inflammatory signaling, and ERS being particularly prominent.

In periprosthetic tissues from patients with AL, TiPs were readily detectable, accompanied by increased expression of IL-1β and iNOS (Fig. 1D to F). In vitro experiments confirmed that TiPs up-regulated IL-1β mRNA levels (Fig. 1G) and promoted its secretion in BMDMs (Fig. 1H). Cellular immunofluorescence demonstrated that TiPs induced ASC expression in macrophages, suggesting inflammasome assembly (Fig. 1I and Fig. S1A). Additionally, TiP stimulation triggered IRF3 nuclear translocation and IFN-β induction (Fig. 1J and K and Fig. S1B), indicating activation of IFN-stimulated genes.

Mounting evidence suggests that ERS and MS are crucial regulators of macrophage inflammation and M1 polarization. Consistent with this, confocal microscopy revealed enhanced MitoTracker fluorescence intensity in TiP-treated macrophages, indicating marked mitochondrial oxidative stress (Fig. 1L). Immunofluorescence staining further showed that ATF6 translocated from the cytoplasm to the nucleus upon TiP stimulation (Fig. 1M and Fig. S1C). Western blot analysis confirmed that TiPs activated key ERS biomarkers, including phosphorylated IRE1 and PERK, at 2 and 4 h poststimulation (Fig. 1N and Fig. S1D and E). These findings collectively demonstrate that TiPs elicit both ER and MS responses, which may synergistically amplify macrophage inflammatory activation.

### DDX3X was identified as the hub gene related to inflammation, ERS, and immune response in TiP-induced macrophages

**Fig. 2.**
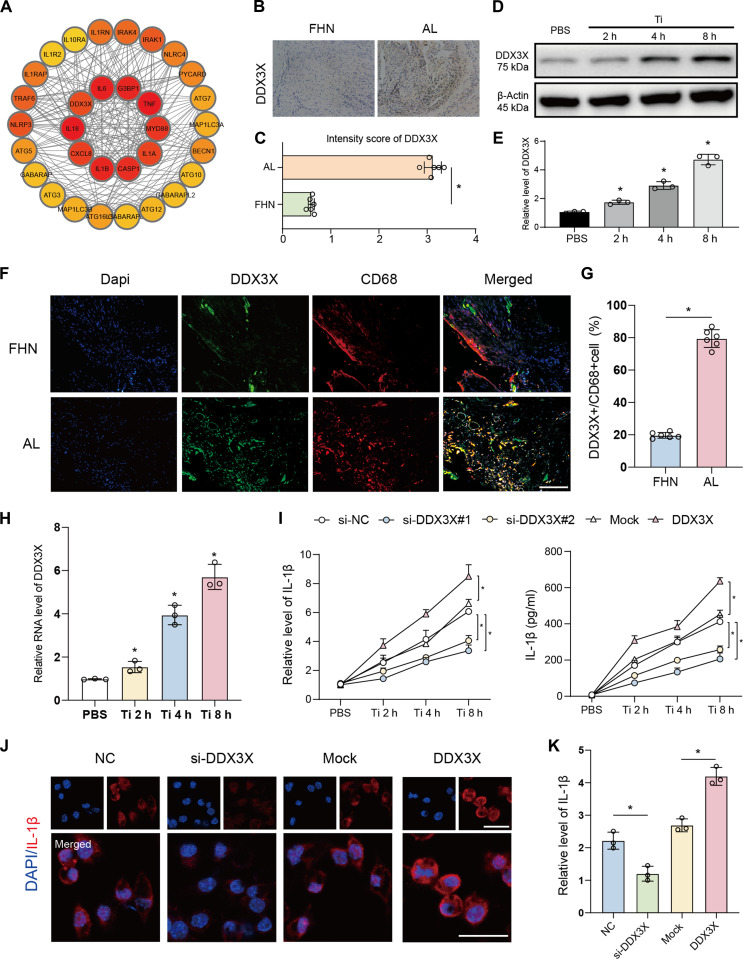
DDX3X was up-regulated in AL synovium and induced IL-1β production. (A) Hub gene network that was generated from DEGs. (B and C) DDX3X was augmented in AL synovium. Representative images of synovium IHC were displayed (B) and intensity score was calculated (C). (D and E) Immunoblot of DDX3X in TiPs-provoked macrophages (D). PBS was set as the control group; other macrophages underwent 2-, 4-, and 8-h TiPs treatment. Quantification of gray value was conducted by ImageJ (E). (F and G) Tissue immunofluorescence of AL and FHN synovium with DDX3X and CD68 antibodies (F). DDX3X and CD68 double-positive cells were calculated by ImageJ (G). (H) mRNA level of DDX3X in macrophages stimulated by TiPs. (I) mRNA and supernatant concentration of IL-1β regulated by DDX3X silencing or overexpression. Macrophages were stimulated by TiPs for 2, 4, and 8 h compared to PBS treatment. (J and K) Cellular immunofluorescence targeting IL-1β was performed to validated DDX3X regulation. Scale bar, 25 μm (J). Intensity of fluorescence was quantified by ImageJ (K). Results displayed in the form of mean ± SD utilized two-tailed Student *t* test, 1-way ANOVA, and 2-way ANOVA for calculating statistical significance. All experiments were triplicated at least. *P < 0.05.

To identify the key regulator of TiP-induced inflammation, we constructed a hub gene network from the DEGs, which included DDX3X (Fig. 2A). We therefore conducted a series of experiments focusing on DDX3X in BMDMs to explore its regulatory role in TiP-induced macrophage inflammation. DDX3X expression was significantly increased in the synovium of patients with AL compared to those with FHN (Fig. 2B and C). Similarly, in vitro, DDX3X protein levels were elevated in a time-dependent manner with prolonged TiP stimulation (Fig. 2D and E). Dual-target immunofluorescence staining for DDX3X and the macrophage marker CD68 in FHN and AL synovial tissues revealed a marked increase in DDX3X^+^/CD68^+^ double-positive cells in the AL group (Fig. 2F and G and Fig. S2A to C), confirming DDX3X up-regulation in macrophages within the AL microenvironment. In vitro experiments further verified that TiPs induced DDX3X up-regulation in BMDMs (Fig. 2H). Functional assays demonstrated that DDX3X knockdown significantly reduced both IL-1β mRNA expression and protein secretion (Fig. 2I). Immunofluorescence analysis of IL-1β confirmed that DDX3X positively regulated IL-1β production in BMDMs (Fig. 2J and K), with DDX3X silencing decreasing IL-1β expression and DDX3X overexpression substantially enhancing IL-1β levels. Collectively, these findings indicate that DDX3X is a key factor in the AL microenvironment and plays a critical role in macrophage-mediated aseptic inflammation.

### DDX3X promotes NLRP3 inflammasome activation and M1 polarization in TiP-stimulated BMDMs

**Fig. 3.**
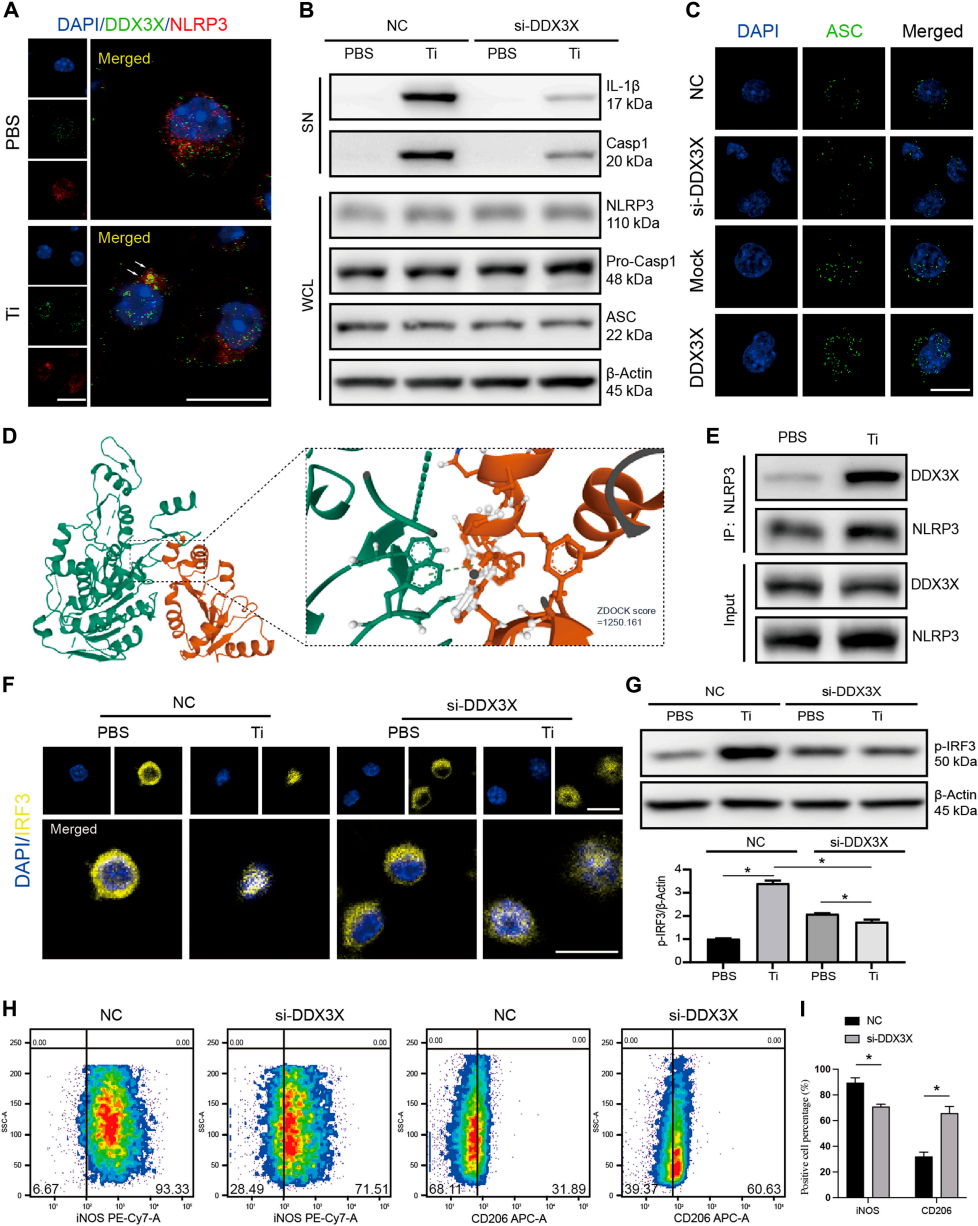
DDX3X regulated TiP-induced macrophages inflammation by interacting with NLRP3 inflammasome. (A) Confocal microscopy images displayed DDX3X and NLRP3 intracellular location. White arrows indicated the DDX3X–NLRP3 complex. Scale bar, 25 μm. (B) Immunoblots of multiple NLRP3 inflammasome-associated markers. SN, supernatant; WCL, whole cellular lysate. (C) ASC expression in DDX3X-regulated macrophages. Scale bar, 25 μm. (D) The predicted molecular docking model of DDX3X and NLRP3. ZDOCK score was displayed. (E) Coimmunoprecipitation and immunoblots displayed DDX3X interacted with NLRP3 in TiP-induced macrophages. (F) Immunofluorescence targeting intracellular IRF3 showed that silencing DDX3X hindered IRF3 entering nucleus. (G) Inhibiting DDX3X reduced the phosphorylation of IRF3 triggered by TiPs. Quantification of immunoblots gray level was conducted with ImageJ. (H and I) Flow cytometry of macrophages transfected with negative control or siRNA targeting DDX3X. Macrophage polarization was assessed with iNOS (M1 marker) and CD206 (M2 marker). Quantitative bar graphs summarize the percentage of M1 and M2 macrophages from independent biological replicates. Results displayed in the form of mean ± SD utilized two-way ANOVA for calculating statistical significance. All experiments were triplicated at least. *P < 0.05.

To explore the mechanism by which DDX3X modulates IL-1β production in TiP-challenged macrophages, we focused on its potential interaction with the NLRP3 inflammasome—a key upstream regulator of IL-1β maturation. Cellular immunofluorescence staining for DDX3X and NLRP3 in PBS- versus TiP-treated macrophages revealed that TiP stimulation significantly enhanced their colocalization (Fig. 3A). Functional assays showed that DDX3X silencing inhibited TiP-induced increases in IL-1β and caspase-1 levels in the supernatant, without affecting intracellular NLRP3, pro-caspase-1, or ASC expression (Fig. 3B). However, immunofluorescence analysis of ASC demonstrated that DDX3X facilitated ASC oligomerization (Fig. 3C). Meanwhile, molecular docking simulations predicted a high-affinity binding interaction between DDX3X and NLRP3, suggesting a potential role in regulating inflammasome assembly (Fig. 3D). Coimmunoprecipitation assays confirmed that TiP stimulation promoted physical interaction between DDX3X and NLRP3 (Fig. 3E). Additionally, DDX3X silencing impaired TiP-induced IRF3 nuclear translocation (Fig. 3F) and phosphorylation (Fig. 3G). Flow cytometry analysis of macrophage polarization markers (iNOS for M1, CD206 for M2) revealed that DDX3X knockdown reduced the proportion of iNOS^+^ cells by 21.45% ± 2.6% (*P* < 0.05) while increasing CD206^+^ cells by 30.06% ± 3.1% (*P* < 0.05). Quantitative bar graphs summarize the percentage of M1 and M2 macrophages from independent biological replicates (mean ± SD) (Fig. 3H and I). Collectively, these findings indicate that DDX3X interacts with the NLRP3 inflammasome and modulates IRF3 activation, thereby regulating macrophage polarization and inflammatory responses in the context of TiP exposure.

### DDX3X regulated mitochondrial injury and ERS in TiP-induced BMDMs

**Fig. 4.**
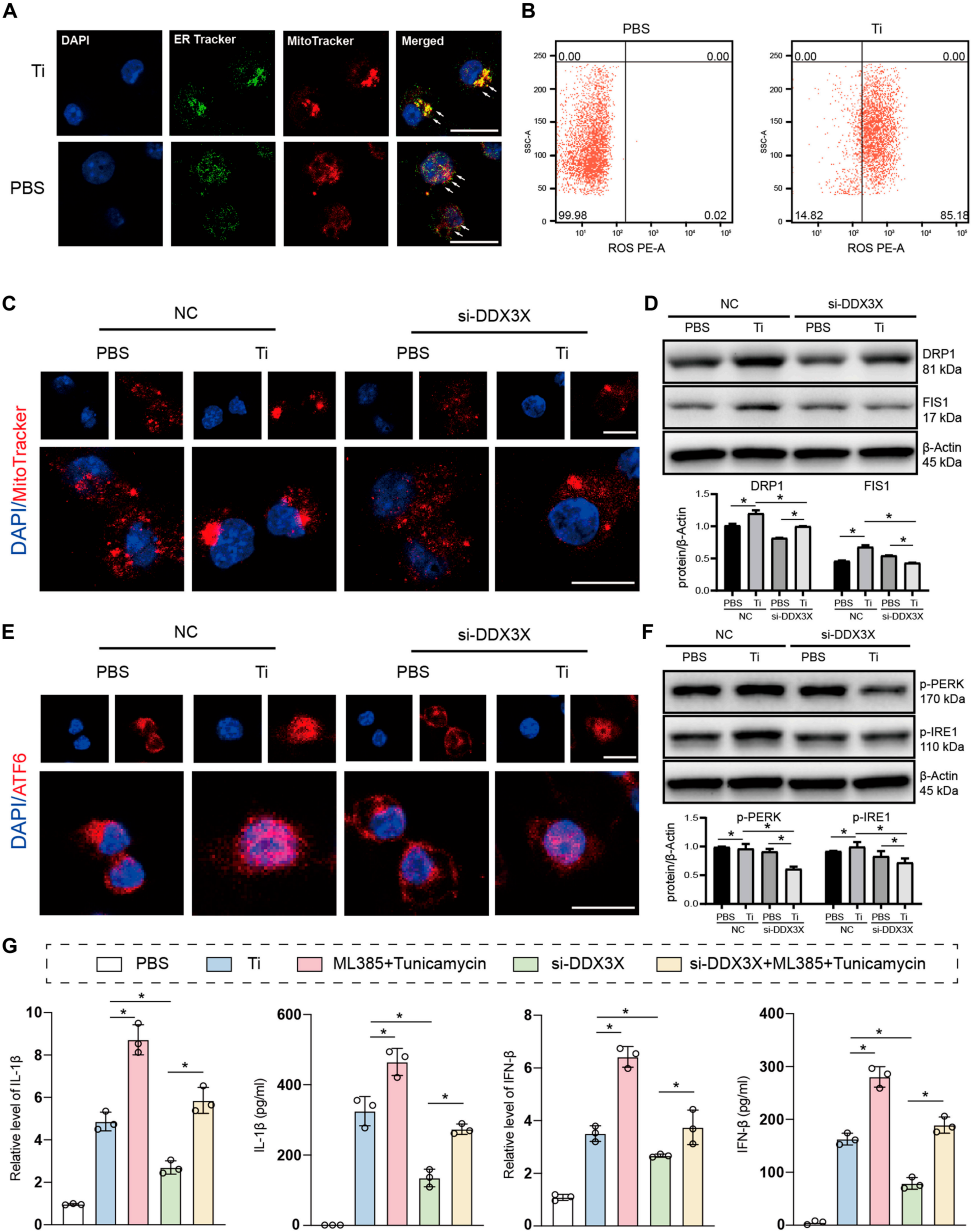
DDX3X regulated TiP-induced Mito–ER interaction. (A) Mitochondria and ER in TiP-induced macrophage. White arrows indicated combinative mitochondria and ER. Scale bar, 25 μm. (B) Flow cytometry displayed TiPs facilitated ROS production in macrophages. (C) Mitochondria staining of TiP-induced macrophages transfected with siRNA-DDX3X and NC. Scale bar, 25 μm. (D) Immunoblots of mitochondria dynamic markers DRP1 and FIS1. Bar plot of gray level was generated by ImageJ. (E) Cellular immunofluorescence targeting ATF6 in macrophages. Scale bar, 25 μm. (F) Immunoblots of ERS markers p-PERK and p-IRE1. Bar plot of gray level was generated by ImageJ. (G) Relative mRNA expression and extracellular secretion of IL-1β and IFN-β for macrophages treated by PBS, TiPs, ML385 + Tunicamycin, si-DDX3X and si-DDX3X + ML385 + Tunicamycin. Results displayed in the form of mean ± SD utilized 1-way ANOVA and 2-way ANOVA for calculating statistical significance. All experiments were triplicated at least. *P < 0.05.

Mitochondrial injury and ERS are critical organellar dysfunctions that drive cellular inflammation. Using ER Tracker and MitoTracker staining, we observed that TiP stimulation significantly enhanced the colocalization of mitochondria and the ER in macrophages, indicating crosstalk between these 2 organelles (Fig. 4A and Fig. S3A to C). ROS—a by-product of mitochondrial respiration—induce oxidative stress and subsequent cellular damage, including the promotion of aseptic inflammation. As detected by a ROS-specific probe, TiP exposure markedly increased intracellular ROS production (Fig. 4B). MitoTracker staining alone revealed that TiPs triggered mitochondrial aggregation, while DDX3X silencing effectively attenuated this aggregation (Fig. 4C). To further explore mitochondrial dysfunction, we analyzed the expression of mitochondrial dynamics markers (DRP1 and FIS1) via immunoblotting: TiPs up-regulated DRP1 and FIS1 expression, and this effect was reversed by DDX3X inhibition (Fig. 4D). We next examined ERS markers (ATF6, p-PERK, and p-IRE1) using cellular immunofluorescence and immunoblotting. Results showed that DDX3X positively regulated TiP-induced ATF6 nuclear translocation (Fig. 4E) and the phosphorylation of PERK and IRE1 (Fig. 4F). To clarify whether mitochondrial dysfunction or ERS contributes to TiP-induced inflammation, we treated macrophages with ML385 (a mitochondrial ROS activator) and tunicamycin (an ERS inducer). Coadministration of ML385 and tunicamycin further enhanced TiP-mediated up-regulation of IL-1β and IFN-β, while DDX3X suppression partially reversed this proinflammatory effect (Fig. 4G). Collectively, these data demonstrate that DDX3X promotes the production of proinflammatory cytokines by regulating mitochondria–ER (mito–ER) crosstalk in TiP-challenged macrophages.

### GATA6 alleviates macrophage inflammation by inhibiting DDX3X transcription

**Fig. 5.**
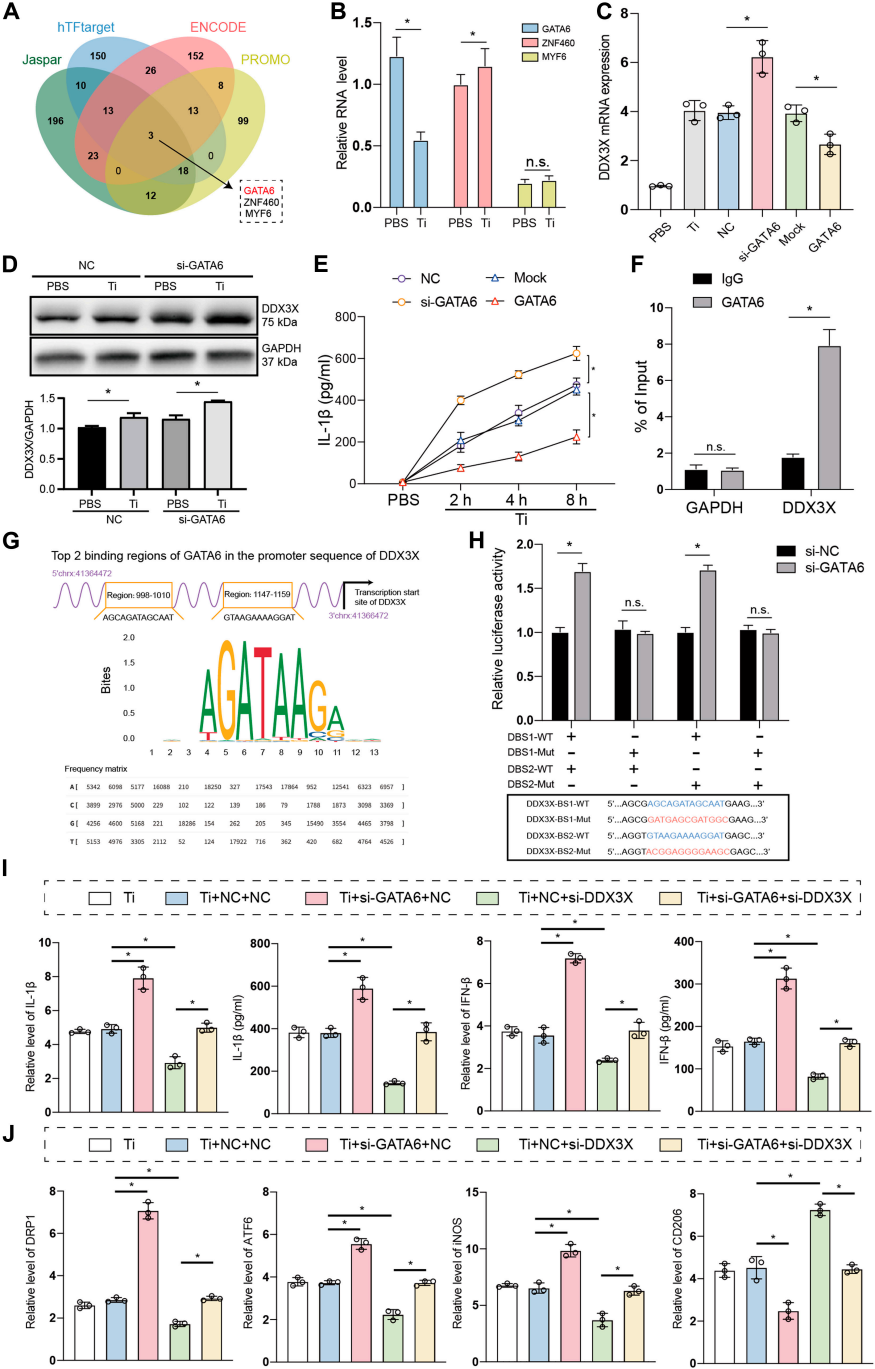
GATA6 transcriptionally regulated DDX3X expression. (A) Venn diagram of predictive DDX3X transcriptional regulator. JASPAR, hTFtarget, ENCODE, and PROMO were utilized. GATA6 was annotated. (B) RT-qPCR of GATA6, ZNF460, and MYF6 in TiP-stimulated bone marrow-derived macrophages (BMDMs). (C) DDX3X mRNA level in siRNA-GATA6/oe-GATA6 plasmid-transfected BMDMs. (D) Representative images of immunoblots targeting DDX3X. Quantification of immunoblots was conducted with ImageJ. (E) Enzyme-linked immunosorbent assay (ELISA) of IL-1β secretion in siRNA-GATA6/oe-GATA6 plasmid-transfected BMDMs. (F) ChIP-qPCR of GATA6-conjugated chromatin DNA. (G) Schematic diagram displaying the 2 TSS binding regions of GATA6 on DDX3X. (H) Dual luciferase reporter assay of GATA6 on DDX3X TSS region. (I) ELISA and RT-qPCR of IL-1β and IFN-β in siRNA-GATA6/siRNA-DDX3X transfected BMDMs. (J) RT-qPCR of DRP1, ATF6, iNOS, and CD206 mRNA level in siRNA-GATA6/siRNA-DDX3X transfected BMDMs. Results displayed in the form of mean ± SD utilized 1-way ANOVA and 2-way ANOVA for calculating statistical significance. All experiments were triplicated at least. *P < 0.05.

To identify the upstream regulator of DDX3X in TiP-stimulated macrophages, we first predicted potential transcriptional factors (TFs) by analyzing data from 4 databases—JASPAR, hTFtarget, ENCODE, and PROMO. This cross-database analysis yielded 3 common candidate TFs: GATA6, ZNF460, and MYF6 (Fig. 5A). To validate the functional relevance of these candidates, we performed in vitro RT-qPCR experiments, which showed that GATA6 was significantly down-regulated in response to TiP stimulation (Fig. 5B)—a finding that prioritized GATA6 for further investigation. We then manipulated GATA6 expression in BMDMs via transfection with either GATA6-specific small interfering RNA (siRNA-GATA6) or a GATA6 overexpression plasmid (oe-GATA6). RT-qPCR results demonstrated that GATA6 markedly inhibited DDX3X mRNA expression (Fig. 5C), and this regulatory relationship was further confirmed by immunoblotting: specifically, silencing GATA6 led to a notable increase in DDX3X protein levels (Fig. 5D). Given that DDX3X is known to modulate IL-1β production, we next measured IL-1β secretion via ELISA. Consistent with DDX3X’s role, we found that GATA6 suppressed IL-1β secretion (Fig. 5E). To test whether GATA6 regulates cytokine production by transcriptionally targeting DDX3X, we performed ChIP-qPCR. This assay confirmed that GATA6 directly binds to the chromatin DNA of the DDX3X promoter (Fig. 5F). To map the precise GATA6-binding site within the DDX3X promoter, we first used the JASPAR database to predict 2 high-likelihood binding regions. We then constructed 4 reporter plasmids carrying either wild-type or mutant versions of the DDX3X promoter and performed dual-luciferase reporter assays. These experiments revealed that GATA6 binds specifically to binding site 1 (but not site 2) in the DDX3X promoter. We further explored the functional consequences of the GATA6/DDX3X axis in TiP-stimulated BMDMs. RT-qPCR and ELISA analyses showed that silencing DDX3X significantly reduced the mRNA expression and secretion of both IL-1β and IFN-β; importantly, cotransfection of si-GATA6 and si-DDX3X reversed this inhibitory effect (Fig. 5I). Similarly, cotransfection of si-GATA6/DDX3X abrogated the TiP-induced suppression of DRP1 and ATF6, indicating that the GATA6/DDX3X axis modulates both mitochondrial and ERS responses. Additionally, phenotypic analysis revealed that GATA6 promotes M2 macrophage polarization (rather than M1 polarization) by inhibiting DDX3X (Fig. S4A and B). Collectively, these findings demonstrate that GATA6 serves as a critical transcriptional regulator of DDX3X in TiP-stimulated macrophages, and further establish the GATA6/DDX3X axis as a key mediator of inflammatory cytokine production, organelle stress, and macrophage polarization.

### Characterization of si-DDX3X NPs@Hy

**Fig. 6.**
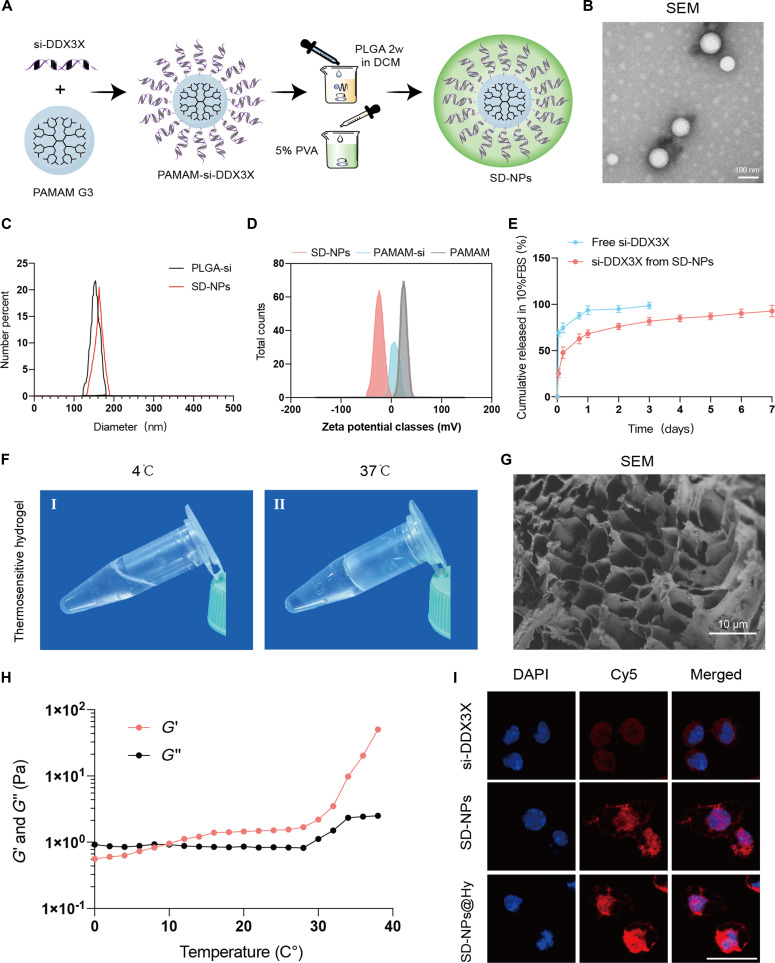
Characterization of SD-NPs and thermosensitive hydrogel. (A) Schematic diagram of siRNA-DDX3X loaded nanoparticles. (B) Representative image of nanoparticles from a scanning electron microscope. Scale bar, 100 nm. (C and D) Diameter (C) and zeta potential (D) of nanoparticles. (E) Cumulative release curve of si-DDX3X from NPs/free si-DDX3X. (F) The thermosensitive feature of hydrogel used. The hydrogel remains liquid at 4 °C and coagulated at 37 °C. (G) Representative image of hydrogel from a scanning electron microscope. Scale bar, 10 μm. (H) *G*′ and *G*ʺ measurement of hydrogel at different temperatures. (I) Confocal microscopic scanning of macrophages treated with sole si-DDX3X, si-DDX3X loaded nanoparticles, and SD-NPs@Hy. Scale bar, 25 μm.

Given that DDX3X modulates mito–ER crosstalk and promotes TiP-induced inflammation, we developed DDX3X-targeting NPs and associated hydrogel formulations to evaluate their translational potential. Small interfering RNA against DDX3X (si-DDX3X) was encapsulated within a PAMAM–PLGA complex (Fig. 6A and B). The average diameter of PLGA-si-DDX3X and SD-NPs was 151.78 ± 3.64 nm and 162.89 ± 4.35 nm, respectively (Fig. 6C). The zeta potential of SD-NPs was –24.58 ± 0.34 mV (Fig. 6D). To quantify the loading capacity, we determined that the siDDX3X loading contents were 15.4 ± 0.7 μg/mg for PLGA-si-DDX3X, 18.1 ± 0.9 μg/mg for PAMAM-si-DDX3X, and 24.6 ± 1.1 μg/mg for SD-NPs (Fig. S5). The hybrid SD-NPs exhibited the highest loading capacity, likely due to the synergistic effect of PLGA encapsulation and PAMAM electrostatic complexation. The encapsulation efficiency (EE%) of SD-NPs was further confirmed to be 82.3% ± 2.4% (Table S3). Cumulative release assays demonstrated that SD-NPs exhibited significant controlled-release efficiency (Fig. 6E). To minimize biotoxicity and reduce clearance rates, an NP-loaded hydrogel (SD-NPs@Hy) was fabricated. The hydrogel showed excellent thermosensitive properties (Fig. 6F) and a microscopic porous structure (Fig. 6G). Rotational rheometry confirmed its sol-gel transition, with the storage modulus (*G′*) exceeding the loss modulus (*G″*) as the temperature increased to 37 °C, indicating stable gel formation (Fig. 6H). Confocal imaging revealed significantly enhanced intracellular uptake of Cy5-labeled siDDX3X in the SD-NPs and SD-NPs@Hy groups compared with free siRNA, with the highest transfection efficiency observed in the SD-NPs@Hy group (Fig. 6I). Based on the measured loading content, the dosage for in vivo experiments was standardized by incorporating 0.8 mg of SD-NPs into each hydrogel injection, ensuring a consistent delivery of approximately 20 μg of siDDX3X per mouse. These results provide robust evidence that siDDX3X was successfully loaded, efficiently delivered, and quantitatively controlled within the SD-NPs@Hy system.

### Si-DDX3X NPs@Hy injection reduced the development of osteolysis in cranial osteolysis mouse models

**Fig. 7.**
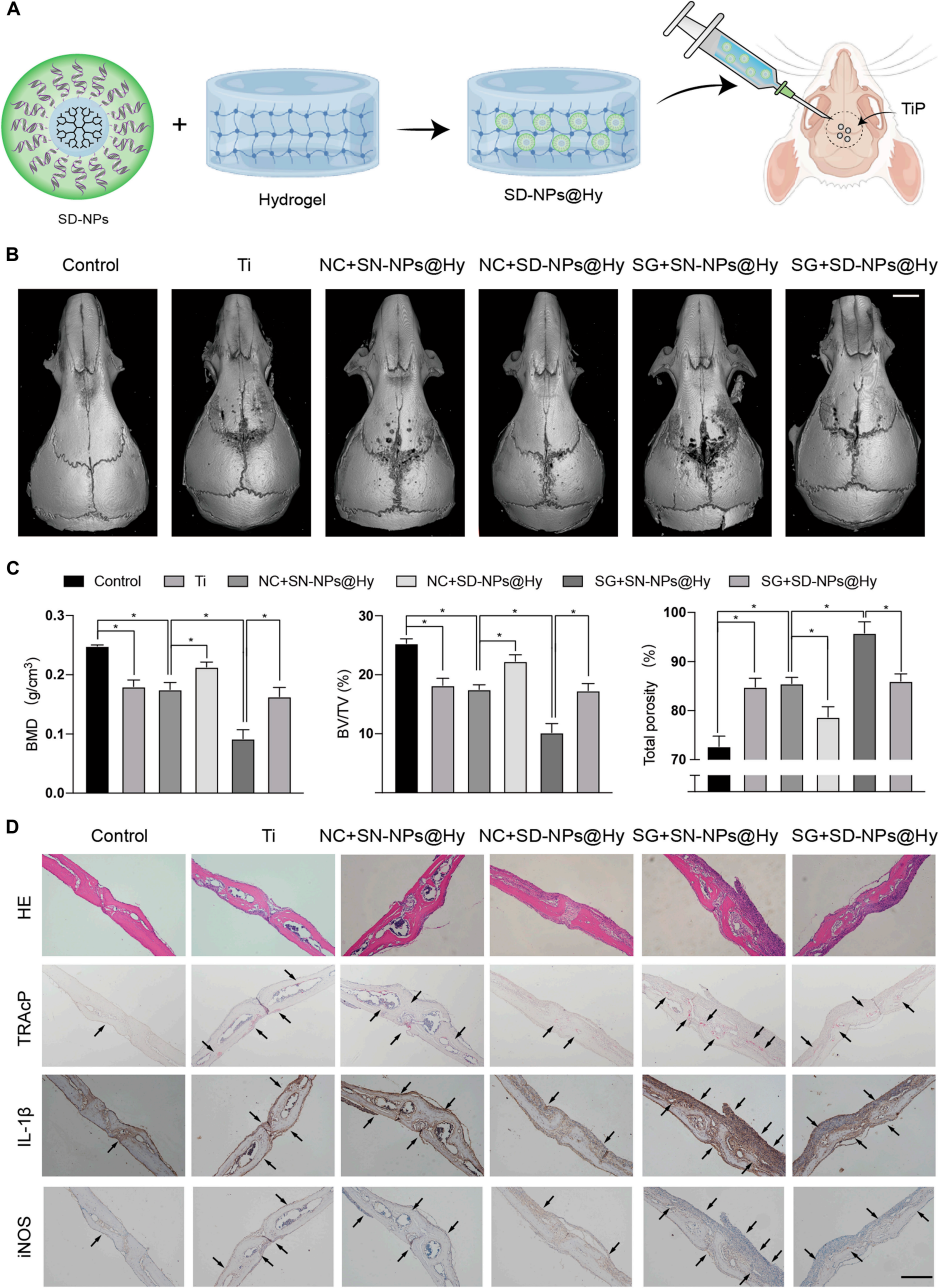
Preparation of SD-NPs@Hy, relieving the cranial osteolysis induced by TiPs. (A) Schematic diagram of SD-NPs@Hy preparation. (B) Micro-CT scanning of the nanoparticle-loaded hydrogel-treated mice cranial osteolytic model. Scale bar, 5 mm. (C) Bone mineral density (BMD), bone volume/total volume ratio (BV/TV), and total porosity were calculated according to micro-CT 3D reconstructed images. (D) HE, TRAcP, and multiple targets’ IHC staining of mice calvaria sections. Scale bar, 250 μm.

In vivo, we evaluated the therapeutic efficacy of the si-DDX3X NPs@Hy formulation. Following preparation of si-DDX3X NPs@Hy, the hydrogel was administered via local injection into a murine model of cranial osteolysis (Fig. 7A). Mouse calvariae were harvested and subjected to micro-CT scanning, with representative 3D reconstructed images presented in Fig. 7B. Treatment with si-DDX3X NPs@Hy significantly alleviated TiP-induced osteolysis, while this therapeutic effect was reversed by coadministration of si-GATA6 lentiviruses. Subsequent histological analyses were performed on sectioned calvarial specimens. Tartrate-resistant acid phosphatase staining (TRAcP) confirmed that TiP stimulation markedly increased osteoclast recruitment. Notably, GATA6 knockdown further promoted osteoclast formation, whereas this effect was suppressed by si-DDX3X NPs@Hy treatment. BMD, BV/TV, and total porosity were calculated by referring to 3D reconstructed images with results showing the similar trends (Fig. 7C). We then conducted immunohistochemical (IHC) staining targeting the proinflammatory cytokine IL-1β, as well as the macrophage polarization marker iNOS. These IHC results demonstrated that the GATA6/DDX3X signaling axis mediates calvarial inflammation (Fig. 7D). Collectively, these in vivo findings identify GATA6/DDX3X as a critical regulatory pathway in TiP-induced osteolysis and highlight the promising therapeutic potential of si-DDX3X NPs@Hy for future clinical translation.

**Fig. 8.**
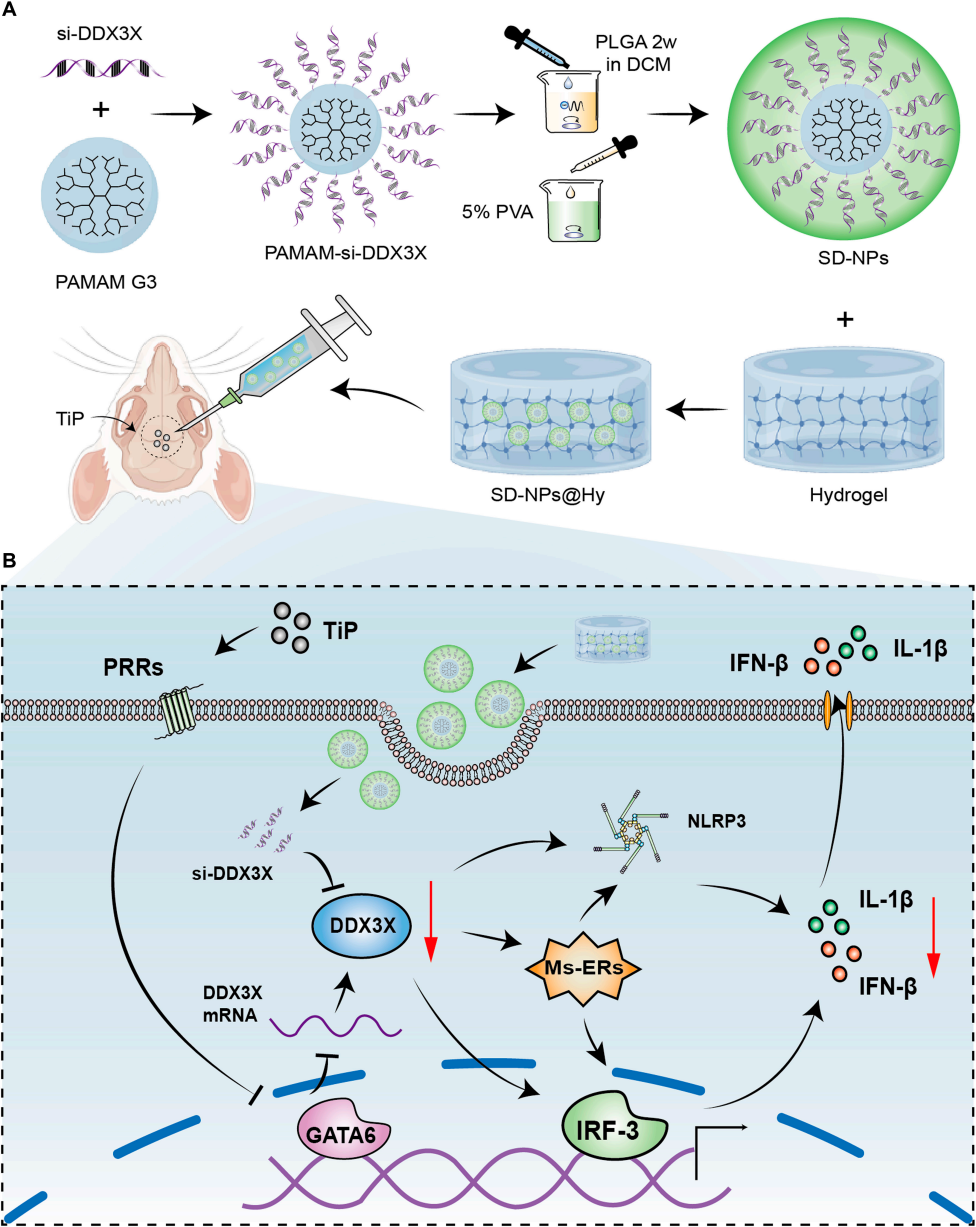
Schematic illustration of the preparation of SD-NPs@Hy and its mechanism in alleviating TiP-induced osteolysis. (A) Synthesis of SD-NPs@Hy and its mode of action in a mouse model of aseptic loosening. (B) Proposed mechanism by which SD-NPs@Hy disrupts GATA6/DDX3X-mediated macrophage inflammation.

Collectively, these in vivo findings identify GATA6/DDX3X as a critical regulatory pathway in TiP-induced osteolysis and highlight the therapeutic potential of si-DDX3X NPs@Hy for future clinical translation. The schematic in Fig. 8 further illustrates the preparation of si-DDX3X NPs@Hy and its proposed mechanism in disrupting GATA6/DDX3X-mediated macrophage inflammation to attenuate osteolysis.

## Discussion

AL remains a critical challenge compromising the long-term success of joint arthroplasty, with titanium (Ti) particle-induced macrophage inflammation as a central pathogenic driver [38]. This study identifies the GATA6/DDX3X axis as a key regulator of Ti particle-mediated inflammatory responses and osteolysis, while demonstrating the therapeutic potential of an NP-loaded injectable hydrogel targeting this pathway.

In our study, we firstly notified that proinflammatory cytokines and pathways were both activated in TiP-induced macrophages and clinical specimens. Abnormal MS and ERS were spotted as well. Subsequently, DDX3X was deemed as a crucial regulator in TiP-challenged macrophages. It facilitated IL-1β secretion and M1 polarization by interacting with NLRP3 inflammasome and regulating mito–ER dynamics. We discovered that GATA6, as a pivotal suppressing TF of DDX3X, uncovers the controlling strategy of DDX3X. Finally, we manufactured si-DDX3X RNA NP-loaded thermosensitive hydrogel, and SD-NPs@Hy displays its exceeding curative effect on TiP-induced osteolysis in vivo.

Our findings ascertained DDX3X as a pivotal hub gene linking Ti particle exposure to macrophage activation, mito–ER reciprocity, and downstream osteolysis. Clinically, DDX3X expression is significantly up-regulated in synovium from patients with AL, particularly colocalizing with CD68^+^ macrophages, highlighting its relevance to AL pathology. Mechanistically, DDX3X promotes NLRP3 inflammasome assembly—evidenced by increased ASC expression and IL-1β secretion—and interacts directly with NLRP3, as demonstrated by coimmunoprecipitation and molecular docking analyses. This interaction likely amplifies inflammasome activation, a well-known driver of proinflammatory cytokine release in periprosthetic tissues. In TiP-induced macrophages, mitochondria interacted with ER, which was never reported before. This mito–ER reciprocity was likely regulated by DDX3X expression. DDX3X facilitated mitochondrial aggregation and ATF6 nuclear translocation induced by TiPs, and impaired mitochondrial dynamics and the expression of ER stress-related markers. Meanwhile, DDX3X could reverse the proinflammatory effect of ML385 and tunicamycin. DDX3X was previously reported as part of stress granule, whose assembly suppressed NLRP3 inflammasome activation [15]. DDX3X had a positive effect on mitochondrial biogenesis and mitophagy [18]. The specific inhibitor of DDX3X, RK-33, could obviously inhibit inflammation and oxidative stress in a sepsis mouse model [39]. DDX3X/NLRP3 signaling has also been reported as one crucial signal triggering pyroptosis in macrophage-related disorders [40]. The data proved the proinflammatory role of DDX3X in TiP-induced macrophages. Titanium wear particles are known to function as endogenous danger signals capable of activating both TLR-mediated inflammatory priming and NLRP3 inflammasome signaling. In line with the classical 2-signal model of NLRP3 activation, TLR engagement provides a priming signal, whereas cellular stress responses serve as the activation signal. In this study, we specifically focused on the DDX3X-dependent regulation of the activation phase of NLRP3 inflammasome signaling. Our data demonstrate that DDX3X governs TiP-induced ERS, mitochondrial dysfunction, and ROS generation, all of which are key drivers of inflammasome assembly in damage-associated molecular pattern (DAMP)-driven sterile inflammation. Thus, DDX3X acts as a critical stress-responsive regulator linking TiP-induced organelle perturbation to NLRP3 inflammasome activation.

Next, the mito–ER interplay in TiP-induced macrophage was discussed. Rowland and Voeltz [41] summarized the function of ER and mitochondria contacts: lipid exchange during biosynthesis, ER control of mitochondrial biogenesis, mitochondrial dynamics and inheritance control, and Ca^2+^ transfer coordination. The connected mitochondria and ER were termed MAMs, and MAMs were critical for inflammasome assembly, antiviral response, and defense of pathogenic bacteria. For example, MAMs provided a platform for pro-caspase-1, ASC, and inactive NLRP3, forming into mature NLRP3 inflammasome [42]. MAMs facilitated STING or Gp78 to interact with MAVS and further triggered interferon response [43]. In our study, TiPs accelerated mitochondria colocalized with ER, which probably formed mito–ER contact sites and MAMs. Since DDX3X could interfere with NLRP3 inflammasome, we subsequently verified that DDX3X was capable of modulating mitochondrial function and control mito-dynamics. For ERS marker ATF6, inhibition of DDX3X could hinder it falling off from ER and entering nucleus. Suppressing DDX3X partially rescues the inflammation caused by ML385 and tunicamycin, suggesting that DDX3X may involve in mito–ER interplay and there are probably other regulators. By targeting DDX3X, we simultaneously dampen both stress pathways, offering a more comprehensive approach than single-cytokine inhibition.

Effective treatment of AL requires localized, sustained modulation of periprosthetic inflammation to avoid systemic immunosuppression. Our si-DDX3X NP-loaded injectable hydrogel (si DDX3X-NPs@Hy) addresses this need through its unique properties: (a) thermosensitivity, enabling liquid-to-gel transition at body temperature for localized retention; (b) controlled release of si-DDX3X, prolonging therapeutic efficacy; and (c) biocompatible formulation, minimizing off-target effects [37]. In vivo, si-DDX3X-NPs@Hy significantly reduces Ti particle-induced calvarial osteolysis, as evidenced by micro-CT and histology—decreasing osteoclast numbers (TRAcP^+^ cells) and proinflammatory markers (IL-1β, IFN-β, and iNOS).

Titanium wear particle-induced inflammation is accompanied by elevated oxidative stress and activation of canonical proinflammatory signaling pathways. ROS have been reported to down-regulate GATA family transcription factors, including GATA6, in inflammatory contexts, and antioxidant treatment can restore GATA6 expression by reducing ROS accumulation, indicating that GATA6 expression is sensitive to redox changes. Antioxidant treatment effects further support the notion that oxidative stress-driven pathways can modulate transcription factor networks, including those governing macrophage identity. Meanwhile, MAPKs and NF-κB are well-established mediators of stress and inflammatory signaling and are activated under oxidative stress conditions, linking upstream stress cues to downstream transcriptional responses. Collectively, these studies provide mechanistic support for the notion that TiP-induced cellular stress converges on GATA6 suppression through redox-sensitive and inflammatory signaling pathways, thereby facilitating macrophage phenotypic reprogramming. Notably, the therapeutic effect of si-DDX3X-NPs@Hy is partially reversed by GATA6 knockdown, confirming that DDX3X is the critical target. This specificity validates the GATA6/DDX3X axis as a therapeutic node and supports the hydrogel’s potential for clinical translation. Compared to systemic drug delivery or surgical debridement, this approach offers site-specific action, reducing risks of systemic toxicity and preserving normal immune function.

While our study provides mechanistic and preclinical insights, limitations exist. First, the precise molecular mechanisms by which DDX3X interacts with NLRP3 and IRF3 warrant further investigation—for example, whether DDX3X modulates their RNA stability or posttranslational modification. Second, long-term studies in large animal models are needed to evaluate the hydrogel’s biomechanical compatibility with bone tissue and its durability in load-bearing joints. Although AL represents a chronic condition, it is increasingly recognized that early inflammatory amplification and macrophage dysregulation play a critical initiating role in driving progressive osteolysis. Therefore, achieving a therapeutically effective concentration of bioactive factors during the acute inflammatory activation phase may be sufficient to reset the inflammatory microenvironment and prevent continual tissue damage. Consistent with this concept, our hydrogel exhibited a rapid-to-stable release phase within the first several days, which coincided with the time window of maximal inflammatory activity. The sustained biological benefit observed in vivo further suggests that short-term functional intervention can result in prolonged immunoregulatory effects. Nevertheless, longer-term release and degradation profiling (4 to 8 weeks) remains an important direction for further translational optimization and will be evaluated in future studies. With regard to the animal models for AL, although the mouse calvarial model successfully reproduces the inflammatory and osteolytic features of wear particle-induced bone destruction, it does not fully replicate the weight-bearing biomechanics and rapid synovial fluid turnover of a true joint replacement environment. Therefore, the current results primarily demonstrate the biological therapeutic effectiveness of the hydrogel rather than confirming its mechanical persistence in load-bearing joints. The thermosensitive hydrogel forms an in situ gel network after injection, which may enhance its local retention; however, dedicated evaluation under dynamic mechanical stress remains necessary. Future work will focus on validating hydrogel stability, retention, and degradation behavior in joint-mimicking mechanical systems or large-animal arthroplasty models to better support clinical translation. Finally, the modification of applied biomedical materials and their loaded therapeutic agents will be further investigated in subsequent studies. Combining DDX3X silencing with pro-osteogenic factors (e.g., BMP-2) could enhance bone repair alongside inflammation suppression, a strategy worthy of exploration. This study is positioned as a mechanism-driven translational work, in which DDX3X is identified as a critical inflammatory regulator and a thermosensitive hydrogel serves as a clinically relevant delivery platform to validate the therapeutic feasibility of locally suppressing DDX3X signaling in TiP-induced aseptic osteolysis. Rather than aiming to develop a new material through stepwise material engineering optimization, we constructed the dendrimer served to stabilize and efficiently deliver DDX3X-siRNA and the hydrogel provided localized and sustained exposure. Future studies will further delineate the incremental contribution of each formulation stage to fully establish an optimized delivery development pathway.

## Conclusion

This study identifies the GATA6/DDX3X axis as a key regulator of Ti particle-induced macrophage inflammation and osteolysis, highlighting DDX3X as a therapeutic target. The NP-loaded injectable hydrogel offers a clinically feasible strategy to locally inhibit DDX3X, mitigating periprosthetic inflammation and improving implant longevity. These findings advance our understanding of AL pathogenesis and provide a foundation for developing targeted therapies to enhance joint arthroplasty outcomes.

## Data Availability

The data that support the findings of this study are available from the corresponding authors upon reasonable request.
