# Supplementary material for: Nanoparticle-Loaded Injectable Hydrogel Alleviates Titanium Particle-Induced Osteolysis by Disrupting GATA6/DDX3X-Mediated Macrophage Inflammation
Source: Biomater Res. 2026 Apr 16;30:0353. doi: 10.34133/bmr.0353 (PMC13084059; doi:10.34133/bmr.0353)
Supplement: Supplementary 1 — Tables S1 to S3 Figs. S1 to S5 [file bmr.0353.f1.docx]

**+Supporting Information**

**Nanoparticle-Loaded Injectable Hydrogel Alleviates Titanium Particle-Induced Osteolysis by Disrupting GATA6/DDX3X-Mediated Macrophage Inflammation**

Sipeng Lin^1,2,3^, Taihe Liu^2^, Qi Zhu^1^, Zhuji Ouyang^2^, Yifan Yu^2^, Haopeng Sun^2^, Changchuan Li^2^, Shixun Li^2^, Chenhao Pan^2^, Wing Cheuk Ko^2^, Haoxian Liu^2^, Jin Liu^1^, Shuangxin Li^1^, Jinchang Chen^1^, Shaojian Wu^1^, Jichao Ye^1,3^, Liangbin Gao^1,2*^, Yue Ding^1,2*^

^1^ Department of Orthopedics, Shenshan Medical Center, SunYat-sen Memorial Hospital, Sun Yat-sen University, Shanwei, Guangdong, P. R. China. 516621.

^2^ Department of Orthopedic Surgery, Sun Yat-sen Memorial Hospital, Sun Yat-sen University, Guangzhou, Guangdong, P. R. China. 510000.

^3^ Guangdong Provincial Key Laboratory of Cancer Pathogenesis and Precision Diagnosis and Treatment，Shanwei, Guangdong, P. R. China. 516621.

* Corresponding author: Yue Ding, [dingyue@mail.sysu.edu.cn](mailto:dingyue@mail.sysu.edu.cn); Liangbin Gao, [gaolb@mail.sysu.edu.cn](mailto:gaolb@mail.sysu.edu.cn).

These authors contributed equally: Sipeng Lin, Taihe Liu, Qi Zhu

**
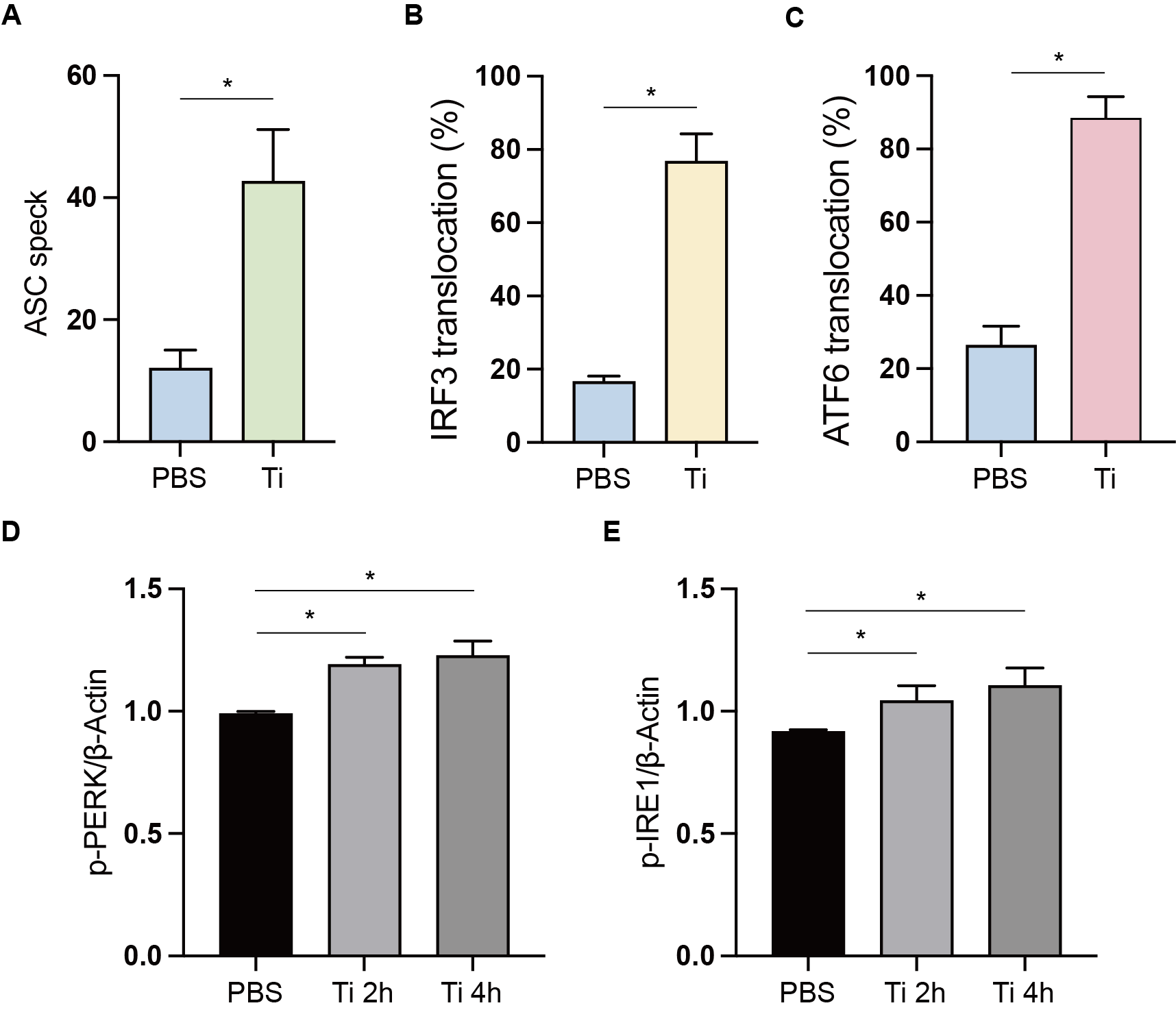
**

Fig S1. (A) Quantification of ASC specks in TiP-stimulated macrophages and control group. (B) Percentage of IRF3 nuclear translocation (C) Percentage of ATF6 nuclear translocation (D-E) Relative expression ratio of p-PERK/β-Actin (D) and p-IRE1/β-Actin (E) by measuring gray level of Western Blot. All experiments were triplicated at least. *p < 0.05.

**
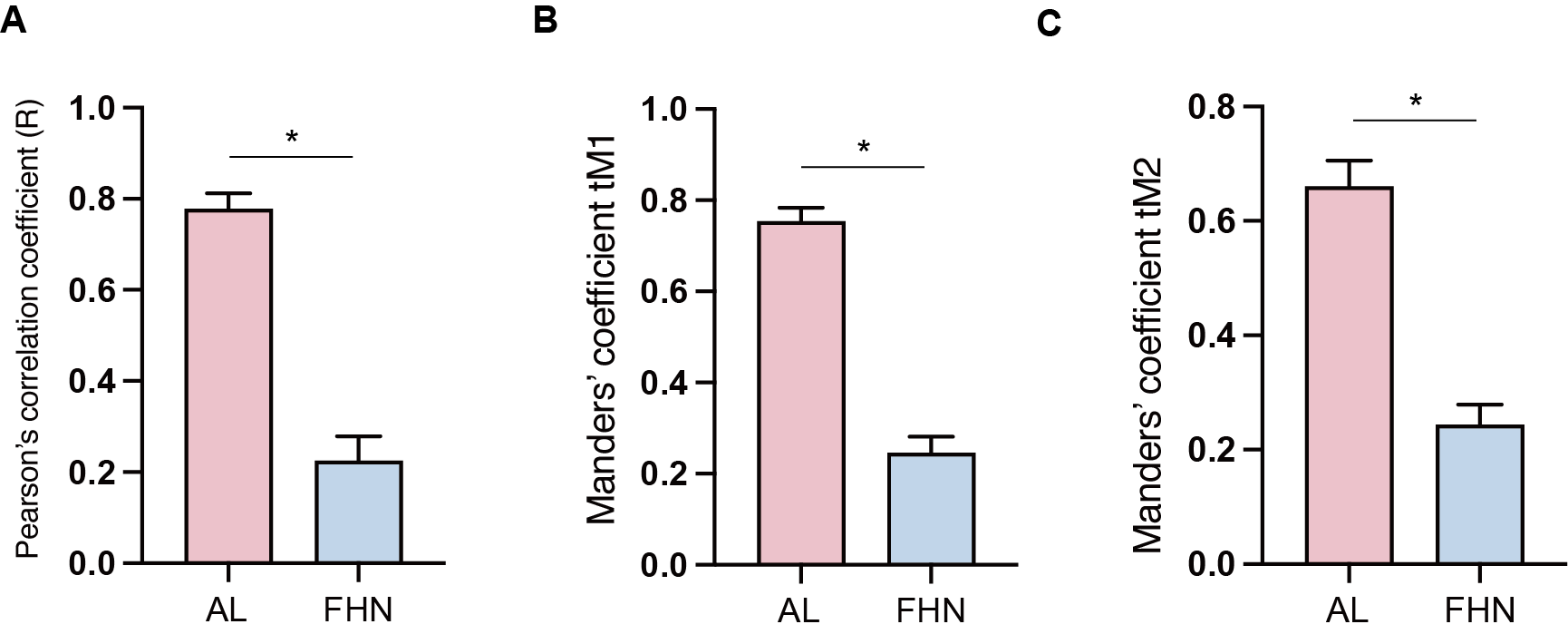
**

Fig S2. (A) Pearson’s correlation coefficient of DDX3X and CD68 in AL and FHN synovium. (B-C) Quantifying the fraction of DDX3X signal overlapping with CD68 (B) and vice versa (C) by Manders’ coefficients. All experiments were triplicated at least. *p < 0.05.

**
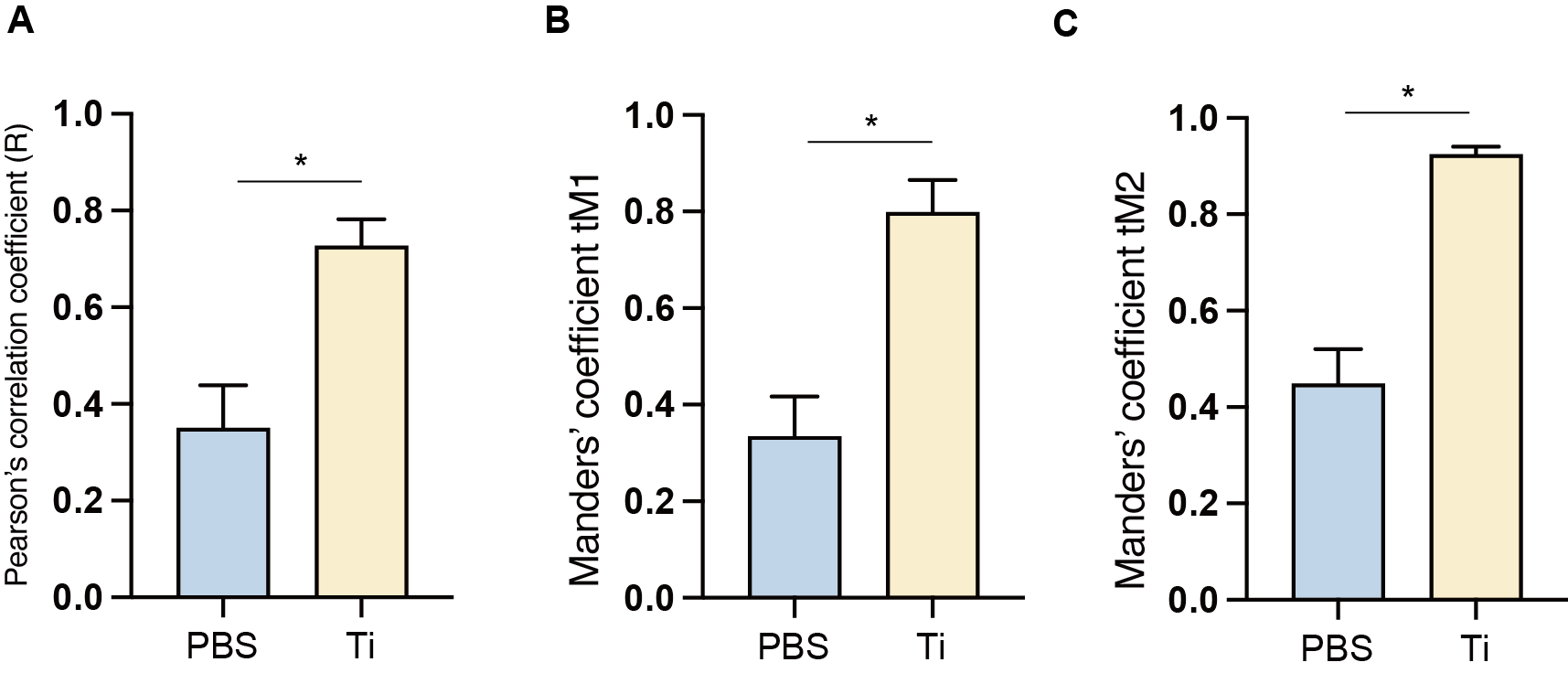
**

Fig S3. (A) Pearson’s correlation coefficient of MitoTracker and ER-Tracker in PBS and TiP-treated macrophages. (B-C) Quantifying the fraction of MitoTracker signal overlapping with ER-Tracker (B) and vice versa (C) by Manders’ coefficients. All experiments were triplicated at least. *p < 0.05.

**
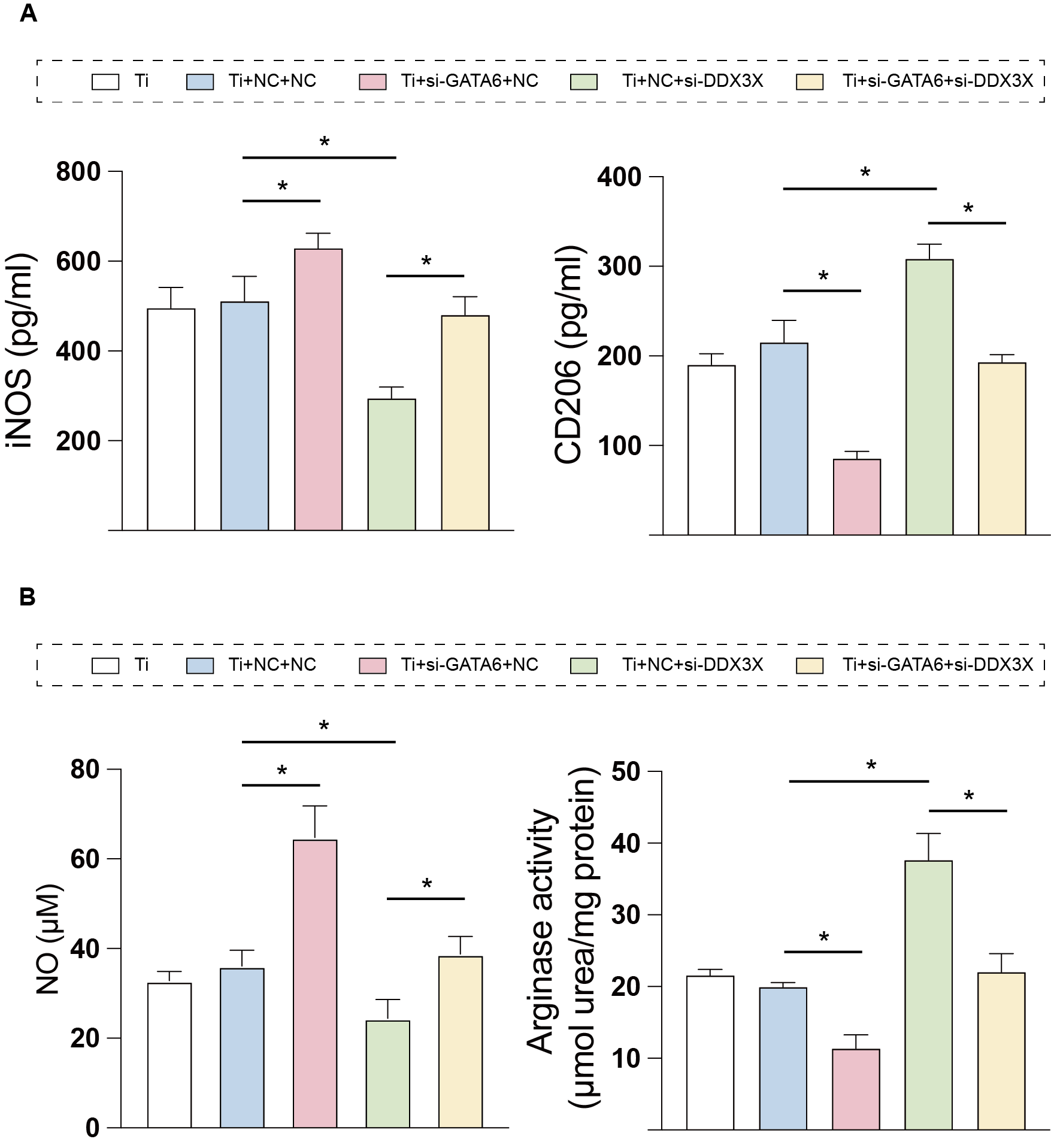
**

Fig S4. (A) ELISA analysis of iNOS and CD206 in GATA6-, DDX3X-, or co-silenced macrophages. (B) nitric oxide (NO) production assays and arginase activity measurements in GATA6-, DDX3X-, or co-silenced macrophages**.** All experiments were triplicated at least. *p < 0.05.


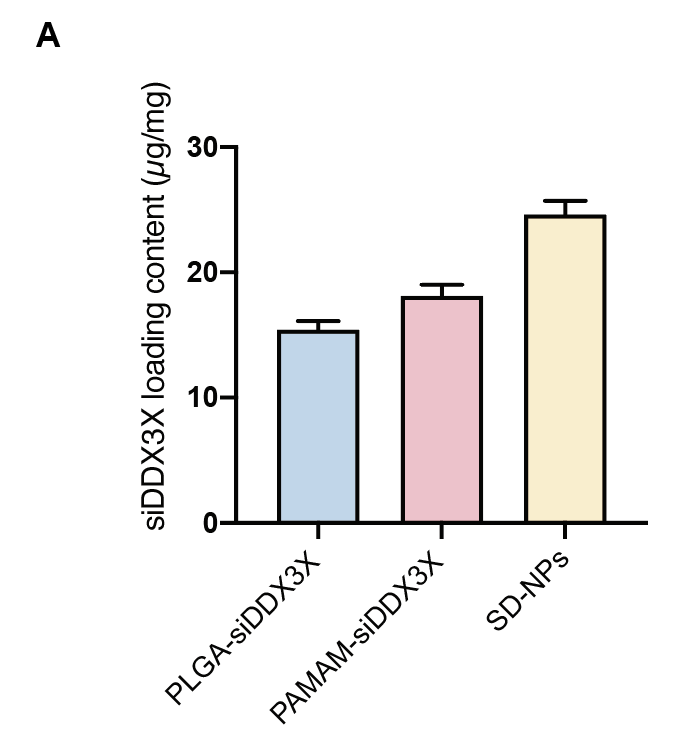


Fig S5. (A) The siDDX3X loading content in PLGA-siDDX3X, PAMAM-siDDX3X, and SD-NPs was quantified and expressed as µg siRNA per mg nanoparticles. Data are presented as mean ± SD (n = 3).

**Table S1. primers for reverse transcription and real-time PCR.**

| Primer names | Sequences (5′- > 3′) |
| --- | --- |
| IL-1β | F: GAAATGCCACCTTTTGACAGTG  R: TGGATGCTCTCATCAGGACAG |
| DDX3X | F: GTAGCAGTCGTGGACGTTCT  R: ACCTGTGTGCCAAGGTTTGA |
| GATA6 | F: CTCAGTTCCTACGCTTCGCAT  R: GTCGAGGTCAGTGAACAGCA |
| DRP1 | F: GCAGAGTAGCGGGAAGAGTT  R: TCCATCCACTCCGTTCTCCT |
| ATF6 | F: CCGATCTTCCCAGATCTTCAG  R: CACCCTGGATGAGGACAACTG |
| iNOS | F: TCACGACACCCTTCACCACAA  R: CCATCCTCCTGCCCACTTCCTC |
| CD206 | F: CACCATCGAGGAATTGGACT  R: ACAATTCGTCATTTGGCTCA |
| GAPDH | F: TGTGTCCGTCGTGGATCTGA  R: TTGCTGTTGAAGTCGCAGGAG |

**Table S2. Sequence of siRNAs in this study.**

| Names | Gene sequences (5’ to 3’) |
| --- | --- |
| DDX3X-siRNA#1 | GGAGGAUUUCUUAUACCAUTT |
| DDX3X-siRNA #2 | AUGGUAUAAGAAAUCCUCCTT |
| GATA6*-*siRNA#1 | GUGGACUCUACAUGAAACUTT |
| GATA6*-*siRNA#2 | GCUCUGGUAAUAGCAAUAATT |

**Table S3. The size, encapsulation efficiency, and zeta potential of the SD-NPs.**

| Names | Average particle size (nm) | Zeta potential (mv) | Entrapment efficiency (%) |
| --- | --- | --- | --- |
| PLGA-si-DDX3X | 151.78 ± 3.64 | / | 70.5 ± 3.2 |
| PAMAM-si-DDX3X | / | 7.08 ± 0.77 | 72.8 ± 2.7 |
| SD-NPs | 162.89 ± 4.35 | -24.58 ± 0.34 | 82.3 ± 2.4 |
